# Perinatal influences on academic achievement and the developing brain: a scoping systematic review

**DOI:** 10.3389/fpsyg.2024.1352241

**Published:** 2024-06-19

**Authors:** Deborah Schneider, Florence Bouhali, Caroline G. Richter, Radu Costache, Catalina Costache, Kaitlyn Kirchhoffer, Vatsa Sheth, Ibo MacDonald, Fumiko Hoeft

**Affiliations:** ^1^Department of Psychological Sciences, University of Connecticut, Storrs, CT, United States; ^2^Webster University, Geneva, Switzerland; ^3^Aix Marseille University, CNRS, CRPN, Marseille, France; ^4^Department of Psychology, University of Alabama at Birmingham, Birmingham, AL, United States; ^5^Institute of Higher Education and Research in Healthcare, Faculty of Biology and Medicine, University of Lausanne, Lausanne, Switzerland; ^6^Department of Psychiatry and Behavioral Sciences and Weill Institute for Neurosciences, University of California, San Francisco, San Francisco, CA, United States; ^7^Department of Neuropsychiatry, Keio University School of Medicine, Tokyo, Japan

**Keywords:** academic achievement, fetal alcohol spectrum disorder (FASD), mathematics, neurodevelopment, neuroimaging, perinatal insults, prematurity, reading

## Abstract

**Introduction and methods:**

In this PRISMA-compliant systematic review, we identify and synthesize the findings of research in which neuroimaging and assessments of achievement have been used to examine the relationships among aspects of developmental programming, neurodevelopment, and achievement in reading and mathematics.

**Results:**

Forty-seven studies met inclusion criteria. The majority examined the impact of prematurity (*n* = 32) and prenatal alcohol exposure (*n* = 13). Several prematurity studies reported a positive correlation between white-matter integrity of callosal fibers and executive functioning and/or achievement, and white matter properties were consistently associated with cognitive and academic performance in preterm and full-term children. Volumetric studies reported positive associations between academic and cognitive abilities and white and gray matter volume in regions such as the insula, putamen, and prefrontal lobes. Functional MRI studies demonstrated increased right-hemispheric language processing among preterm children. Altered activation of the frontoparietal network related to numerical abilities was also reported. Prenatal alcohol exposure studies reported alterations in white matter microstructure linked to deficits in cognitive functioning and academic achievement, including mathematics, reading, and vocabulary skills. Volumetric studies reported reductions in cerebral, cerebellar, and subcortical gray matter volumes associated with decreased scores on measures of executive functioning, attention, working memory, and academic performance. Functional MRI studies demonstrated broad, diffuse activation, reduced activation in canonical regions, and increased activation in non-canonical regions during numeric tasks.

**Discussion:**

A preponderance of studies linked prematurity and prenatal alcohol exposure to altered neurodevelopmental processes and suboptimal academic achievement. Limitations and recommendations for future research are discussed.

**Systematic review registration:**

Identifier: DOI 10.17605/OSF.IO/ZAN67.

## Introduction

Reading and arithmetic computation are learned behaviors; however, the neurocognitive scaffolding supporting them is constructed before birth, in infancy and in childhood (Raffington et al., 2021). The biological component of this structure is principally encoded in the DNA, but its expression is shaped by the prenatal and postnatal environments. This shaping process is characterized as *developmental programming*, or the contribution of environmental factors to the biology, behavior, and epigenome of the organism (Langley-Evans, 2006). Recent research has explored the relationship between developmental programming, academic achievement, and educational attainment, drawing explicit links between developmental programming, cognitive abilities and their neurobiological correlates (e.g., Räikkönen et al., 2011; Bijnens et al., 2019; Lammertink et al., 2021).

Numerous factors affect early developmental programming and subsequent physiological and behavioral outcomes; these include maternal and infant nutrition, pre-and post-natal medical conditions, pre-natal exposure to teratogens, pre-and postnatal exposure to toxicants, maternal medical conditions, maternal drug use, maternal alcohol consumption, and maternal psychosocial stress (Langley-Evans, 2006).

### Purpose and research questions

Reading and mathematics achievement is foundational to educational and occupational attainment (Marks, 2006), and vulnerability to learning difficulties in reading and mathematics may be influenced by a complex mix of genetic and specific environmental factors and their interactions (Mascheretti et al., 2013; Kershner, 2020a,b). In the present scoping systematic review, we identify, examine, and synthesize the findings of research in which neuroimaging and assessments of achievement have been used to evaluate and understand the relationships among developmental programming, neurodevelopment, and achievement in reading and mathematics. The principal aims of the present review are to provide an accurate and accessible overview of the major findings of literature in the field and address the following research questions: (1) What is the state of the evidence concerning the relationships among developmental programming, neurodevelopment and achievement in reading and mathematics? (2) What trends can be observed in the literature? (3) What gaps exist in the literature? (4) What are some appropriate directions for future research?

### Rationale

While numerous systematic reviews have examined the relationship between developmental programming and academic achievement (e.g., Van Handel et al., 2007; Williams and Ross, 2007; Irner, 2012; Behnke et al., 2013; Viteri et al., 2015; Yeoh et al., 2019), to our knowledge, no systematic review has synthesized the results of studies using neuroimaging to explore the neurobiological correlates of these relationships and better understand the underlying mechanisms mediating the impact environmental factors on academic achievement. Because the relationships between environmental factors and achievement in reading and mathematics have been amply explored in other research (*Ibid.*), we have turned our attention to those studies including both measures of achievement and neuroimaging.

It would be impossible to identify and examine all the factors contributing to developmental programming in a single review. We therefore include only studies examining high-incidence factors, which we have divided into three broad categories:

1. Child-centric factors, inclusive of prematurity, perinatal asphyxia, and hyperbilirubinemia (jaundice);
2. Maternal factors, inclusive of substance use; hypertensive diseases, including (pre)eclampsia; and maternal infection;
3. Exogenous factors, inclusive of exposure to heavy metals and polyhalogenated hydrocarbons.

We recognize that the boundaries among these categories are porous and that there may be collinearity among factors. Moreover, certain conditions (e.g., prematurity), are often of unknown etiology. Nevertheless, we feel that this categorical framework is useful, providing an organizational structure for our search procedures. As we are interested in the effects of early developmental programming, we have limited our search studies focused on developmental insults from the prenatal to postnatal periods (conception to two-weeks postnatal). Because our interest lies in the relationship between developmental programming and achievement in reading and mathematics, we have included only studies with measures of reading and/or mathematics achievement.

## Method

This scoping systematic review was guided by the principles outlined in the *Cochrane Handbook for Systematic Reviews of Interventions* (Higgins et al., 2019), the *Preferred Reporting Items for Systematic reviews and Meta-Analyses* (PRISMA; Page et al., 2021), and the PRISMA Extension for Scoping Reviews (PRISMA-ScR; Tricco et al., 2018). The study protocol was pre-registered in the Center for Open Science database (Schneider-Richardson et al., 2021). Our design was modified after registration; specifically, we adopted the PRISMA-ScR Checklist (*Ibid.*) as it became clear that our systematic review would be scoping in nature.

### Information sources and search strategy

To identify potential articles, eight electronic databases were searched: APA PsychArticles (American Psychological Association, 2022a), APA PsychInfo (American Psychological Association, 2022b), EBSCO Academic Search Premier (EBSCO Publishing, 2022), Embase (Elsevier, 2022a), ERIC (Institute of Education Sciences, 2022), MedLine (National Library of Medicine, 2022), PubMed (National Center for Biotechnology Information, 2022), and Scopus (Elsevier, 2022b).

Terms were combined using Boolean operators. Filters for journal articles were applied, and searches were limited to title, abstract and keyword and subject terms. Searches were structured to return articles including terms (at least one term per group) from each of four groups: (A) achievement construct, (B) neuroimaging technique, (C) perinatal risk factor, and (D) pre-to post-natal period. See Supplementary Table S1 for search terms.

### Data screening procedures

Search records were uploaded to Covidence (Veritas Health Innovation, 2022); duplicates were removed following machine screening and verification by the first author. The article screening process was blinded using Covidence. Article screening was performed by six student researchers and the article’s first author. Training of student researchers was performed by the article’s first author, including 4 hours of training prior to screening and ongoing training of 1 hour per week for 18 weeks. See Supplementary Information S1 for further details of the screening process. To be retained for review following screening, articles must have satisfied all the following criteria:

1 Publication Criteria.

i Peer-reviewed articles published in English in a (inter)nationally-circulated academic journals between 1 January 1970 to 1 January 2022.

a Books, book chapters, dissertations, white papers, and other manuscripts were excluded.

2 Design Criteria.

i Original research using a quantitative or mixed-methods design.

a Qualitative research, reviews, syntheses, and meta-analyses were excluded.

ii Measures included neuroimaging of any type delineated in Supplementary Table S1 and at least one measure of achievement in reading and/or mathematics.

a Reading measures must have evaluated reading, decoding, comprehension, and/or interpretation of natural written language (inclusive of passages, sentences, phrases, words, syllables, blends, pictographs, ideographs, logographs, graphemes, and alphabet letters/non-alphabetic characters, dependent on the target language’s writing system).

b Mathematics measures must have evaluated arithmetic, mathematic, computational, and/or numeric skills or processes.

iii Analyses included descriptive and/or inferential statistics for at least one measure.

3 Participant Criteria.

i Human subjects with confirmed or suspected exposure to a key perinatal risk factor, aged >/= 36 months at the time of the final evaluation of reading or mathematics achievement (to exclude infant gaze studies and measures of pre-academic skills).

### Measured variables and strategy for data synthesis

We determined the number of studies generated by our search, the number of duplicates removed, the number of articles excluded at the title and abstract and full-text review phases, and the number of articles retained for systematic review. Retained studies’ findings were summarized and coded for key perinatal insult; key achievement construct (reading, math, both); study design (case–control study, cohort study, cross-sectional study, RCT); participant demographics (sample size, sex, age, country, minority status, and socio-economic status [SES]); imaging (modality, task paradigm); achievement instrument (standardized, non-standardized); and findings (significant differences in achievement, significant differences in brain measurements, significant association between achievement and brain measurements).

### Evaluation of study quality

We evaluated article quality using items adapted from the. National Heart, Lung, and Blood Institute (NHLBI)/National Institutes of Health (NIH) (National Heart, Lung, and blood institute, National Institutes of Health, 2013) instruments for the *Quality Assessment for Observational Cohort and Cross-Sectional Studies* and for the *Quality Assessment Case–Control Studies* (2013) and additional items developed by the review’s first author. See Supplementary Information S2 for items and details of the evaluation process. Results are presented in Supplementary Table S2. We elected not to consider effect size as a quality indicator. Our rationale for this choice is explained in Supplementary Information S3.

## Results

### Search results

**Figure 1:**
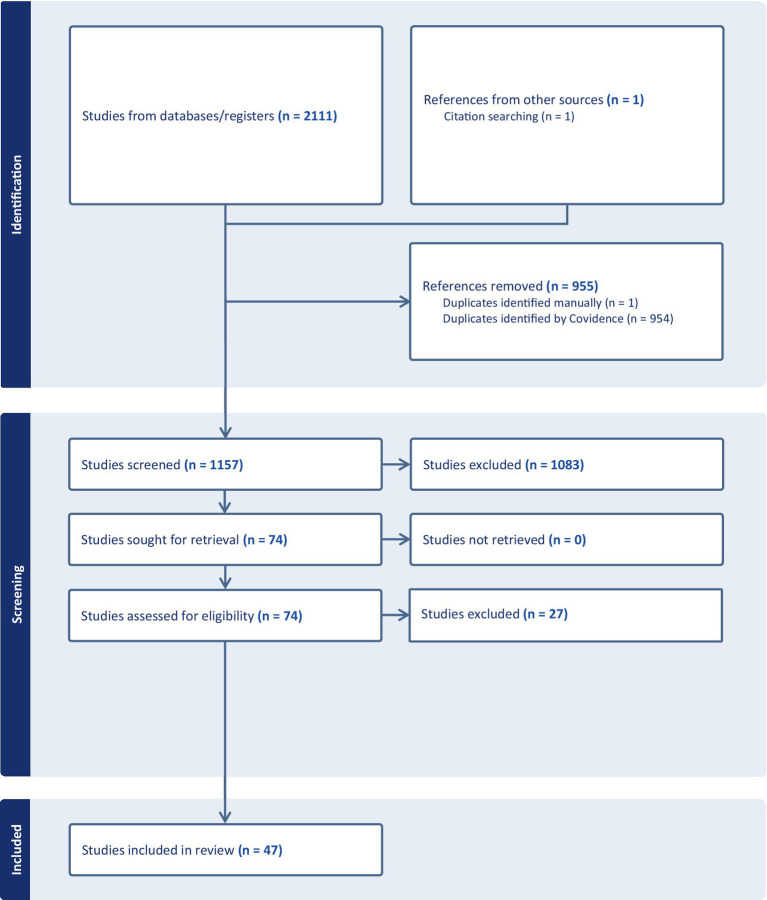
PRISMA flow diagram.

Of the 2,111 studies imported to Covidence, 955 duplicates were removed. Of the remaining 1,156 studies, 1,083 were excluded as irrelevant following title and abstract screening, with a proportional inter-rater agreement of 0.97. The remaining 73 studies were subjected to full-text review of which 49 were retained, with a proportional inter-rater agreement of 0.89. Following full-text screening, three articles were excluded as the authors reported using achievement tests but did not report results. While no formal process of reference chasing was undertaken, one article meeting inclusion criteria was added following article screening as it was explicitly referenced in an included study and was clearly relevant to our review (see Figure 1: PRISMA flow diagram; Page et al., 2021).

### Study characteristics

Our search yielded a total of 47 studies meeting inclusion criteria. These examined the relationship between prematurity (*n* = 32), prenatal alcohol exposure (*n* = 13), perinatal cocaine exposure (*n* = 1), and perinatal organohalogen exposure (*n* = 1); brain development; and achievement in reading and/or mathematics. As only one study each was retained for the insults of perinatal cocaine exposure and perinatal organohalogen exposure, a decision was taken to exclude these studies from the broader review; details of these studies are available in Supplementary Informations S4, S5. Given the variability in study design, measures, and data analyses, we elected to approach the synthesis qualitatively. In the following sections, we report the results of this synthesis by perinatal insult and imaging modality. A summary of study characteristics is displayed in Table 1. Additional details of study characteristics can be found in Supplementary Table S3.

**Table 1:** Characteristics of studies retained in this systematic review.

**First author, date**	**Nation**	**Key insult**	**Achievement construct**	**Study design**	**Imaging modality**	**Imaging paradigm/Output measure**	**Sample size**	**Number of groups**	**Mean age at last reported date**	**% female across groups**	**% minoritized across groups**	**Differences in achievement between/among groups?**	**Association(s) between achievement and imaging?**
**Alcohol studies**													
Ben-Shachar et al. (2020)	South Africa	Alcohol	Math	Cohort study; Case–control	EEG	Task: arithmetic calculation	48	4	16.26	43.73	100.00	Yes	Yes
Gautam et al. (2015)	United States, South Africa	Alcohol	Math	Cohort study; Case–control	sMRI	Structural: volumetric	139	2	12.30	44.60		Yes	Yes
Glass et al. (2017)	United States	Alcohol	Reading, Math	Case–control	sMRI	Structural: morphometric	42	2	15.08	30.95	42.86	Yes	Yes
Lebel et al. (2010)	Canada	Alcohol	Math	Cross-sectional	dMRI	Structural: FA	21	1	9.20	42.86		Not applicable	Yes
Little et al. (2018)	Canada	Alcohol	Reading, Math	Case–control	fMRI	Resting State	133	2	12.60	41.98	40.77	Not applicable	Yes
McLachlan et al. (2020)	Canada	Alcohol	Reading, Math	Cohort study; Case–control	sMRI	Structural: volumetric	139	2	12.70	54.69		Yes	Yes
Meintjes et al. (2010)	South Africa	Alcohol	Math	Case–control	fMRI	Task: addition and number proximity judgment	33	2	10.26	56.51	33.00	Yes	Yes
Miles et al. (2021)	South Africa	Alcohol	Math	Cohort study; Case–control	sMRI	Structural: volumetric	52	3	11.16	82.02	28.46	Yes	Yes
Santhanam et al. (2009)	United States	Alcohol	Math	Cohort study	fMRI	Task: subtraction	54	3	23.17	64.80		Yes	Yes
Sowell et al. (2008)	United States	Alcohol	Reading	Cross-sectional	dMRI	Structural: FA	36	2	10.90	52.78	51.96	Yes	No
Treit et al. (2013)	Canada	Alcohol	Reading, Math	Longitudinal	sMRI, dMRI	Structural: volumetric, MD	44	2	11.77	40.52	28.84	Yes	Yes
Woods et al. (2015)	South Africa	Alcohol	Math	Cohort study	fMRI	Task: addition and symbolic number comparison	49	3	10.31	56.92	40.51	Yes	Yes
Woods et al. (2018)	South Africa	Alcohol	Reading, Math	Case–control	fMRI	Task: non-symbolic number comparison	27	3	11.68	70.36	27.00	No	Yes
**Opioid studies**													
Landi et al. (2017)	United States	Cocaine	Reading	Cohort study	EEG	Task: reading	110	2	17.11	45.45	84.99	Yes	Yes
**Organohalogen studies**													
Margolis et al. (2020)	United States	PBDE	Reading	Cohort study	fMRI	Resting state	33	1	5.00	54.50		Not applicable	Yes
**Prematurity studies**													
Arhan et al. (2017)	Turkey	Prematurity	Mathematics	Case–control	sMRI	Structural: volumetric	46	2	9.15	54.35		Yes	Yes
Allin et al. (2001)	United Kingdom	Prematurity	Reading	Case–control	sMRI	Structural: volumetric	117	2	14.90	46.15		No	Yes
Anderson et al. (2017)	Australia	Prematurity	Reading, Math	Cohort study	sMRI	Structural: morphometric	168	1	7.00	48.00		Not applicable	Yes
Andrews et al. (2010)	United States	Prematurity	Reading	Cohort study; Case–control	dMRI	Structural: FA	28	2	12.16	32.14		Yes	Yes
Bäuml et al. (2017)	Germany	Prematurity	Math	Cohort study; Case–control	fMRI	Resting State	143	2	26.60	38.28		Yes	Yes
Belfort et al. (2016)	Australia	Prematurity	Reading, Math	Cohort study	sMRI	Structural: volumetric	180	1	7.00	51.11		Not applicable	Yes
Bruckert et al. (2019)	United States	Prematurity	Reading	Cohort study; Case–control	dMRI	Structural: FA	71	2	6.15	47.89		No	Yes
Cheong et al. (2013)	Australia	Prematurity	Reading, Math	Cohort study; Case–control	sMRI	Structural: volumetric	280	2	18.00	55.34		Yes	Yes
Clark et al. (2017)	United States	Prematurity	Math	Cohort study; Case–control	fMRI	Task: symbolic and non-symbolic number comparison	20	2	20.07	60.00	40.00	Yes	Yes
Collins et al. (2019)	Australia	Prematurity	Reading, Math	Cohort study; Case–control	dMRI	Structural: FA, RD, MD, neurite density	150	2	13.22	46.67		Yes	Yes
Collins et al. (2021)	Australia	Prematurity	Math	Cohort study; Case–control	dMRI	Structural: fiber density & cross-section (FD, FC, FDC)	236	2	5.71	49.49		Yes	Yes
Dubner et al. (2019)	United States	Prematurity	Reading	Cohort study; Case–control	dMRI	Structural: FA& MD	78	3	6.20	51.27	17.84	No	Yes
Feldman et al. (2012)	United States	Prematurity	Reading	Cohort study; Case–control	dMRI	Structural: FA	42	2	12.77	54.78		No	Yes
Frye et al. (2009)	United States	Prematurity	Reading	Case–control	MEG	Task: reading (phonological)	31	3	16.41	45.17		Yes	Yes
Frye et al. (2010)	United States	Prematurity	Reading	Cohort study; Case–control	dMRI, sMRI	Structural: FA & RD	32	3	16.10	31.00		Not applicable	Yes
Gozzo et al. (2009)	United States	Prematurity	Reading, Math	Case–control	fMRI	Task: Story listening	78	2	9.05	49.85	52.38	Yes	Yes
Isaacs et al. (2001)	United Kingdom	Prematurity	Reading, Math	Cohort study; Case–control	sMRI	Structural: morphometric	36	4	15.75			Yes	Yes
Kelly et al. (2016)	Australia	Prematurity	Reading, Math	Cohort study; Case–control	dMRI	Structural: FA, neurite dispersion, & neurite density	178	2	7.52	51.19		Yes	No
Kesler et al. (2006)	United States	Prematurity	Reading	Cohort study; Case–control	sMRI	Structural: volumes and gyrification	106	2	8.98	47.17		No	Yes
Klein et al. (2014)	Austria	Prematurity	Math	Cross-sectional	fMRI	Task: symbolic number comparison and font size comparison	15	1	7.20	73.33		Not applicable	Yes
Klein et al. (2018)	Austria	Prematurity	Math	Cross-sectional	fMRI	Task: symbolic number comparison and font size comparison	16	1	7.20	75.00		Not applicable	Yes
Ment et al. (2006)	United States	Prematurity	Reading, Math	RCT	fMRI	Task: passive listening	71	2	9.07	49.30	17.26	Yes	Yes
Myers et al. (2010)	United States	Prematurity	Reading	Cohort study; Case–control	fMRI	Task: auditory & visual word-picture matching	67	2	16.00	49.25		Yes	Yes
Pavlova et al. (2009)	Germany	Prematurity	Math	Case–control	sMRI	Structural: morphometric	27	3	14.54	40.74		Yes	No
Scott et al. (2011)	United Kingdom	Prematurity	Reading	Cohort study; Case–control	sMRI	Structural: volumetric	311	2	15.13	0.44		Yes	Yes
Stewart et al. (1999)	United Kingdom	Prematurity	Reading	Cohort Study; Case–control	sMRI	Structural: inspection for neurological abnormalities	93	2	14.90	48.39		Yes	Yes
Thompson et al. (2015)	Australia	Prematurity	Reading, Math	Cohort study; Case–control	dMRI	Structural: FA, MD, AD	92	2	7.55	52.09		No	Yes
Thompson et al. (2020)	Australia	Prematurity	Reading, Math	Cohort study; Case–control	dMRI	Structural: apparent fiber density within tracts of interest	102	2	7.60	54.88		No	No
Travis et al. (2015)	United States	Prematurity	Reading	Cohort study; Case–control	dMRI	Structural: FA	45	2	12.84	51.14		Yes	Yes
Travis et al. (2016)	United States	Prematurity	Reading	Case–control	dMRI	Structural: FA	45	2	12.84	51.27		No	Yes
Ullman et al. (2015)	Australia	Prematurity	Math	Cohort study; Case–control	sMRI, dMRI	Structural: morphometric; FA	240	2	5.81	40.74		Not Applicable	Yes
Van Ettinger-Veenstra et al. (2017)	Sweden	Prematurity	Reading	Cohort study; Case–control	fMRI	Task: reading tasks (orthographic, phonological & semantic judgment)	26	2	13.25	57.70		Yes	Yes

### Prematurity studies

Globally, over 10% of infants are born PT, and the proportion of PT infants has increased substantially since 1990 (Blencowe et al., 2012; Harrison and Goldenberg, 2016), particularly in the developing world with prematurity rates approaching 20% in some nations. Infants born premature or preterm (>/= 37 weeks of gestation), are at increased risk of morbidity and mortality compared to full-term peers, with risk increasing with the degree of prematurity (Manuck et al., 2016). Moreover, they are more likely to experience deficits in language, attention, working memory, and executive functioning, all key correlates of academic achievement (Lee and Park, 2016; Pérez-Pereira et al., 2017; Loeb et al., 2020). Additionally, PT children are more likely to have a diagnosis of a specific learning disorder and to receive special education (Cherkes-Julkowski, 1998; Kirkegaard et al., 2006).

We identified a total of 32 PT studies meeting inclusion criteria. These were published between 1999 and 2021, with 16 (50%) published in 2015 or thereafter. Thirteen (40.63%) were conducted in the United States, followed by Australia (9; 28.13%), the United Kingdom (4; 12.50%), Austria (2; 6.25%), Germany (2; 6.25%), Sweden (1; 3.13%) and Turkey (1; 3.13%). Twenty-one (65.63%) employed cohort designs with case–control. Another six (18.75%) used case–control designs alone; two (6.25%) used cohort designs without a control or comparison group; two (6.25%) were cross-sectional; and one (3.13%) constituted a randomized controlled trial. Four (12.5%) had a single group, and 23 (71.86%) had a two-group design, typically with a matched comparison group. The remaining five (15.63%) divided participants into three or more groups. Eleven (34.38%) used diffusion-weighted/tensor magnetic resonance imaging (dMRI); ten (31.25%) used volumetric, morphometric, or other gross structural (T1) MRI techniques (sMRI); eight (25%) used fMRI; two (6.25%) used both dMRI and sMRI; and one (3.13%) used magnetoencephalography (MEG). Finally, eight studies (25%) employed a task performance paradigm; one (3.13%) involved resting-state image acquisition; and the remaining 23 (71.86%) used structural measurements.

Mean sample size was approximately 100 (99.06; range 15–311). Mean age of participants at the time of the last reported measurement was 11.77 years; an average of 47% of participants were identified as female. Only 12.5% included information on participants’ race or ethnicity and 62.5% on socio-economic status (SES). Over 40% (43.75%) evaluated achievement in reading, 21.8% in mathematics, and 31.35% in both. Just over 90% used standardized achievement instruments, while the remaining studies employed novel measures. Of the 27 studies with a control/comparison group, 18 (66.67%) reported decrements in achievement among PT participants; however, this may be misleading, as groups were often matched for achievement, cognitive abilities, or other factors. Furthermore, all but three studies (90.63%) reported a significant association between achievement and neuroimaging measures among PT participants, FT participants, or both.

Except for the previously referenced weakness in reporting race/ethnicity, the studies generally conformed to quality indicators, as determined by consensus among the research team. All but one (96.88%) documented participant consent/assent and inclusion criteria. All included a description of study aims or goals and research hypotheses or a description of exploratory objectives. Study analyses were judged appropriate for all studies; outcome variables were adequately defined; and findings were reported clearly and consistent with the studies’ designs. Obtained probability values and measures of error or variability were provided for all but one study (96.88%). Moreover, all but one study (96.88%) included both PT and comparison participants, to account for changes that might be attributable to the normal course of development in the human brain (Bethlehem et al., 2022). An overview of study findings, organized by primary imaging modality, is provided below.

#### dMRI studies

Several studies reported significant associations between white matter properties in the corpus callosum (CC) and academic achievement among PT participants, those with low birth weight (LBW; a rough proxy for prematurity), or FT controls. Andrews et al. (2010; *n* = 28), for example, reported significant positive associations between birthweight (among PT and FT children) and fractional anisotropy (FA) in various callosal subregions, and significant positive associations between FA in the genu and body of the CC and reading decoding performance, regardless of birth weight. Similarly, Dubner et al. (2019; *n* = 78) reported significantly lower FA and significantly higher mean diffusivity (MD) in several CC segments among PT participants with neonatal inflammatory conditions relative to FT and PT participants without a history of such conditions. Among all groups, FA in the occipital segment of the CC was significantly associated with performance on measures of cognitive abilities, executive function, and reading.

Thompson et al. (2015; *n* = 92), detected numerous significant differences between 7-year-old PT and FT participants in measures of volume, FA, and diffusivity in subsections of the CC as well as significant negative associations between gestational age (GA) and CC abnormalities. Moreover, white matter microstructural anomalies and delayed development of the CC were associated with poorer performance on evaluations of math achievement, motor skill, and visual perception among PT participants. In a large longitudinal study, Collins et al. (2021; *n* = 236) also reported a significant positive relationship between the rate of maturation of the posterior body of the CC and math achievement. Other findings included positive associations between math achievement and fiber density, fiber-bundle cross-section, and combined fiber density and cross-section in the visual, sensorimotor, and cortico-thalamic/thalamocortical white matter tracts among participants in both groups.

White matter fiber tracts other than the CC were also evaluated. Thompson et al. (2020; *n* = 102) reported significant associations between white matter fiber density in the corticostriatal (CS) and thalamocortical (TC) tracts and reading and motor performance. Reduced fiber connectivity in CS and TC tracts was observed in very preterm (VPT) children, though no link was found between reading achievement and connectivity in the VPT group. In FT controls, reading achievement correlated negatively with connectivity between the left caudate, putamen, and lateral prefrontal cortex, indicating potential differences in neurodevelopmental trajectories between groups.

Frye et al. (2010; *n* = 32) evaluated the relationships among reading-related skills, attention, and micro-and macro-structural properties of white-matter tracts connecting the frontal and posterior brain regions, specifically the superior longitudinal fasciculus (SLF) and the superior/inferior fronto-occipital fasciculus (S/IFOF), in PT and FT 16-year-olds. Lower FA and higher RD in the left SLF were linked to better reading and phonological skills across all participants, consistent with the tract’s well-established involvement in reading, while right-hemisphere SLF FA was linked to inattentiveness. By contrast, the relationship between SLF volume and these skills differed across groups: SLF volume was positively associated with phoneme reversal performance and attention in preterm adolescents but showed the opposite association for attention in full-term adolescents. Moreover, preterm participants exhibited specific macrostructural, but not microstructural, white-matter abnormalities linked to reading skills and cognitive deficits.

Travis et al. (2016; *n* = 45) also reported differences in the relationships between reading performance and fascicular microstructure in PT vs. FT children. Significant group differences were detected in mean FA for the right anterior SLF and the left arcuate fasciculus (AF), but not in their homologues in the CS tract, uncinate fasciculus or inferior longitudinal fasciculus (ILF). While authors detected significant associations between word reading and comprehension and white matter properties in every region but the ILF, these correlations were positive in the PT group and negative in the FT group, again reflecting potential differences in trajectories of neurodevelopment.

Bruckert et al. (2019; *n* = 71), by contrast, did not detect differences between FT and PT children in mean FA of the left AF, bilateral SLF, or the left inferior cerebellar peduncle (ICP), nor did they detect significant differences in reading scores at age eight. However, white-matter characteristics of the left AF, bilateral SLF, and left ICP at age six were associated with reading achievement at age eight among FT participants, suggesting that children born PT may rely upon different pathways than those born full term when developing reading skills.

Travis et al. (2015; *n* = 45) also reported significant associations between white-matter properties within the cerebellar peduncles and reading achievement in both PT and FT children: FA values of the superior cerebellar peduncles (SCP), the middle cerebellar peduncles (MCP), and the bilateral ICP were significantly associated with performance on measures of decoding and reading comprehension among participants across groups; however, correlations were positive for the MCP and negative for the S/ICP. While the direction of association for each region of interest (ROI) was consistent between groups, patterns of association varied, with significant associations between performance on measures of reading comprehension and FA in the right SCP and MCP among FT participants and significant associations between performance on measures of reading comprehension and FA in the left ICP and MCP among PT participants.

Whole-brain imaging constituted another common approach. Kelly et al. (2016; *n* = 177), for example, identified clusters of voxels with lower FA and higher axon dispersion in VPT children compared to controls. No significant differences in axon density were found between groups. Gestational age correlated positively with FA, while neonatal brain abnormality scores correlated negatively with FA and positively with axon dispersion. Higher FA values correlated positively with IQ and academic performance, and negatively with behavioral/emotional problems in VPT children. Axon density was positively correlated with IQ and negatively correlated with behavioral and emotional problems. Although axon dispersion showed a positive correlation with behavioral and emotional problems in a small area, no correlation was detected between axon dispersion or density and academic achievement.

Collins et al. (2019; *n* = 150) used dMRI to examine the associations between whole-brain white-matter microstructure and performance on tasks of reading and mathematics among VPT and FT 13-year-old participants. Among VPT and FT participants, mathematics performance was positively associated with FA and negatively associated with RD and MD, widely across white matter. Higher neurite density was also significantly associated with performance in mathematics, notably in the corona radiata, external capsule, and CC. Furthermore, among VPT children with mathematics impairment, neurite density was particularly reduced.

Analyses of the whole-brain FA skeleton by Feldman et al. (2012; *n* = 42), by contrast, detected statistically-significant positive associations between FA characteristics and measures of decoding, language processing speed, syntactic comprehension, and verbal intelligence among PT participants. The authors reported significant positive correlations between performance on language and reading measures and FA in the CC, forceps minor, bilateral inferior longitudinal fasciculus, right anterior thalamic radiation, right CST, and right inferior fronto-occipital fasciculus. Moreover, FA in the left IFOF and left SLF was positively associated with performance on all language and reading tasks except reading comprehension.

##### Summary

Collectively, the PT microstructure studies present several notable trends. Most prominent is evidence of a significant relationship between white-matter microstructural properties and cognitive and academic performances across various measures, including reading decoding, mathematics achievement, cognitive abilities, and executive function (Andrews et al., 2010; Thompson et al., 2015; Dubner et al., 2019; Collins et al., 2021), consistent with the findings of research in other populations (Qiu et al., 2008; Van Eimeren et al., 2008; Tsang et al., 2009). Most studies reported positive correlations between cognitive and academic functioning and FA in particular, suggesting that better performance was supported by higher myelination, axonal density or axonal coherence. Many such associations were not specific to the PT population but seen along the continuum of abilities in PT and FT individuals.

Additionally, several studies reported findings underlining the essential role of the CC in cognitive functioning and academic performance among preterm and full-term participants. Andrews et al. (2010), Thompson et al. (2015), and Dubner et al. (2019), variously linked CC white-matter properties, including FA and MD, to executive functioning and achievement in reading and mathematics among study participants. Kelly et al. (2016) and Collins et al. (2021) extended these findings, reporting significant associations between CC maturation rate, fiber density, and fiber-bundle cross-section and mathematics achievement and broader cognitive abilities, including IQ. These findings reflect a growing consensus concerning the CC’s implication in academic performance and cognitive function, particularly among PT individuals (Paul, 2011; Thompson et al., 2015).

Specific alterations of neurodevelopmental trajectories were found in PT children, as evidenced by differential associations between white matter properties and task performance between PT and FT participants (Travis et al., 2016; Bruckert et al., 2019; Thompson et al., 2020). Thompson et al. (2020), for example, reported a negative association between reading achievement and connectivity in tracts linking the left caudate and putamen with the lateral prefrontal cortex among FT controls, but not among PT participants. Such results point to altered neurodevelopmental processes in PT children. This pattern is congruent with the body of literature suggesting that early life adversity, such as preterm birth, may lead to the recruitment of alternative neural pathways in affected individuals (Johnson and Marlow, 2017).

#### sMRI studies

Volumetric and morphometric analyses were also used to evaluate the relationship between tissue properties and measures of achievement. In a large-scale study, Ullman et al. (2015; *n* = 240) detected a significant association between tissue volume in the insula and putamen, measured at term-equivalent age by deformation-based morphometry, and mathematics achievement (at five and seven) and working memory (at seven) in PT participants but not in controls. Likewise, neonatal FA was positively associated with both mathematics achievement and working memory in the VPT group at age five.

In another large-scale study, Scott et al. (2011; *n* = 311) used volumetric analyses to examine the relationship between white and grey matter volumes and performance on tests of reading, spelling, verbal fluency, and executive functioning (EF). The relationships between total gray matter volume and test performance differed between groups only for spelling. Specifically, bilateral gray matter volume in the prefrontal lobes (with local maxima in the left medial frontal and right superior frontal gyri) was positively associated with spelling achievement among controls but negatively associated among VPT participants. Laterality and main effects of gender were also detected, with stronger associations between spelling performance and gray matter volume in the left medial frontal cortex extending to the caudate nucleus among female VPT participants compared to female controls and weaker associations among male VPT participants compared to male controls. The opposite pattern was observed for associations between spelling scores and gray matter volume in the left middle frontal gyrus, with stronger associations in VPT male participants compared to male controls and weaker associations in VPT female participants compared to female controls.

Cheong et al. (2013; *n* = 280), in another large-scale study, performed whole-brain volumetric analyses, reporting significant differences between VPT and FT in the volume of all brain structures measured, including cortical white matter, cortical grey matter, thalamus, basal ganglia, cerebellum and hippocampi. Moreover, they found that total brain volume accounted for between 20.4% (math) and 40.5% (reading) of the difference in performance between groups and for 31.9% of the difference in IQ; however, they reported no differences in associations between groups. Volumetric analyses performed by Allin et al. (2001; *n* = 117) produced complementary findings. The authors reported that VPT participants had significantly smaller cerebella than those born at FT. Furthermore, they detected a strong association between cerebellar volume and cognitive abilities, executive functioning (mental processing), working memory, and reading skills among VPT participants. It should be noted that these associations were not detected among FT participants; however, FT data sets were incomplete, lacking intelligence and mental processing test data.

Unique among volumetric studies, Belfort et al. (2016; *n* = 180) examined the association between breastfeeding and brain volume, cognitive abilities, academic achievement in reading and math, language, visual perception, and attentional skills in a cohort of preterm children with VLBW. Results demonstrated a significant positive association between days breast milk consumption (>50% of enteral intake as breast milk) and deep nuclear gray matter volume at term-equivalent age, and performance in IQ, mathematics, working memory, and motor function tests at age seven.

Others used morphometric measurements to detect associations between neuroanatomy and cognitive abilities or achievement. Kesler et al. (2006; *n* = 106) reported that PT participants had increased gyrification bilaterally in the temporal lobe compared to FT controls. Moreover, this increased gyrification was associated with decreased left temporal gray matter volumes and poorer reading recognition scores in PT children, suggesting abnormal cortical development and folding of the temporal lobe (Evaluation of language and reading was not performed for full-term participants). Anderson et al. (2017; *n* = 168) reported statistically significant negative correlations between global brain, cerebral white matter, and deep gray matter abnormality and performance on measures of intelligence, math computation, and motor skills among school-age children born PT.

Arhan et al. (2017; *n* = 46) reported significant reductions in the area of the CC and in the volumes of the cerebellum and hippocampus (but not in whole brain volume) amongst PT children at age 9 compared to age-matched term controls. Notably, these reductions were associated with lower scores across evaluations of cognitive abilities and executive function. Of relevance to this review, they observed a significant positive correlation between CC area and performance on a test of arithmetic reasoning. Likewise, Isaacs et al. (2001; *n* = 36) reported a statistically-significant relationship between morphometric features of the brains of VLBW adolescents and performance in math reasoning and numeric operations. Preterm adolescents with significant discrepancies between arithmetic skills and IQ exhibited smaller gray matter probability in the left intraparietal sulcus compared to PT adolescents with no IQ-arithmetic discrepancy. However, no significant differences were detected between PT adolescents with and without IQ-math reasoning discrepancies.

Pavlova et al. (2009; *n* = 27) analyzed periventricular white-matter lesions and tissue loss (indexed by ventricular volume) associated with periventricular leukomalacia (PVL), with respect to mathematics achievement in PT adolescents with and without PVL and FT controls. Significant decrements in math calculation were observed among PLV PT participants compared to non-PLV PT and FT adolescents. However, the calculation abilities of PVL patients were not linked to the volumetric extent of lesions in right or left parieto-occipital, temporal and frontal regions.

The study performed by Stewart et al. (1999; *n* = 93) was the oldest included in the present review, and its findings constituted an outlier in the data set. While the authors reported decrements in reading age and a much higher rate of abnormal neurological findings (specifically, abnormalities of ventricles, CC, and white matter) among VPT participants, they did not detect a clear relationship between structural abnormalities and measures of achievement or cognitive abilities. Given the advances in imaging technology in the last quarter century, it is possible that the limitations in imaging quality contributed to the outlier status of this study.

##### Summary

Taken together, the findings from PT volumetric and morphometric studies support the hypothesis that neurostructural differences in PT individuals are strongly tied to cognitive and academic outcomes. Despite considerable variation in methodology and imaging techniques, a few notable trends emerged: Most large-scale studies reported significant positive associations between measures of regional and total brain volume and academic and cognitive performance in PT participants. Associations were noted at the whole-brain level (Cheong et al., 2013), and more specifically between white and grey matter volume in regions including the insula, putamen, prefrontal lobes, cerebellum, and basal ganglia, and measures of math achievement, spelling, reading, intelligence, working memory, and executive function (Scott et al., 2011; Ullman et al., 2015). These results align with neurobehavioral research underscoring the enduring effects of prematurity on cognitive and academic performance (Aarnoudse-Moens et al., 2009).

Several PT volumetric and morphometric studies have highlighted the complex relationship between brain structure and cognitive performance and achievement. The studies by Isaacs et al. (2001) and Kesler et al. (2006) collectively indicated that subtle deviations in brain morphology, such as cortical gyrification and gray matter probability, may have significant implications for cognitive performance and academic achievement among PT individuals. These findings align with research in other populations, demonstrating the significant influence of cortical folding patterns on cognitive function (Gregory et al., 2016). Other studies pointed to group differences in the relationship between brain morphometry and cognitive/academic outcomes (Allin et al., 2001; Scott et al., 2011; Ullman et al., 2015), indicating that neurodevelopmental trajectories are influenced by prematurity.

#### fMRI studies

Analysis of functional activations and connectivity constituted another common technique among PT studies. As an example, Gozzo et al. (2009; *n* = 78) used an fMRI language paradigm and tests of intelligence and academic achievement to investigate the relationships between functional connectivity, academic performance, and cognitive abilities among PT and FT participants. PT participants scored significantly lower on measures of cognitive abilities, reading comprehension, and expressive vocabulary. Moreover, functional connectivity analyses demonstrated significantly stronger connectivity of Wernicke’s area with the right inferior frontal gyrus (IFG), and left and right supramarginal gyri among PT participants. Myers et al. (2010; *n* = 67) reported similar results using a passive language task paradigm in the fMRI. Wernicke’s area was more strongly connected to the right supramarginal gyrus in the PT group, with stronger connectivity associated with poorer performance on tests of vocabulary and verbal intelligence (correlations were not reported for reading tasks). Together, these studies indicate that PT children may recruit the right hemisphere, and specifically supramarginal gyrus, to a greater extent for language processing.

A small-scale fMRI study by Van Ettinger-Veenstra et al. (2017; *n* = 26) reported altered neural activations in VLBW children, compared to controls with a normal birthweight, during fMRI reading tasks implicating orthographic, phonological, or semantic judgment. Although there were no significant differences in task performance between groups, VLBW participants showed increased activation in the left IFG during phonological tasks compared to controls, suggesting potential compensatory mechanisms. They also displayed decreased activation in the right supramarginal gyrus and the left IFG during orthographic and semantic tasks, respectively, compared to controls.

Several studies used fMRI and evaluations of numeric skills to explore the neurobiological correlates of mathematics achievement in PT participants. Clark et al. (2017; *n* = 20) conducted a small study of healthy, high-functioning young adults born PT and FT controls. Participants performed a symbolic and non-symbolic magnitude comparison task in the fMRI. Non-symbolic comparison elicited typical activations of the number processing network, including superior and inferior frontal regions and bilateral intraparietal sulci (IPS), and deactivations of the default mode network in all participants. Despite similar task performance, PT individuals showed greater activations than controls in the right inferior frontal cortex and IPS. Elevated signal change was linked to poorer performance on the math fluency task, suggesting difficulties in approximate number processing and compensatory mechanisms in PT.

In a small cross-sectional study, Klein et al. (2014; *n* = 15) used fMRI to examine the neurofunctional correlates of performance on a magnitude comparison task and a Stroop task (i.e., identifying the larger of two numbers based on font size) amongst children born PT. Neural activations to these two tasks largely overlapped in the frontoparietal number-processing network; however, a shift from frontal to parietal activations was observed with increasing GA and birth weight. As this frontoparietal shift in the number processing network is characteristic of typical development, this may suggest a maturational delay in the numerical network of low GA and LBW children. Likewise, participants with lower overall cognitive abilities and/or mathematics achievement displayed more distributed activation in the frontal–parietal network. In a follow-up study based on the same paradigm and data, Klein et al. (2018; *n* = 16) investigated the relationship between GA and the neural processes associated with intentional and automatic number processing. Automatic number processing, measured using a Stroop-like number perception task, was linked to areas of the brain involved in cognitive control, such as the anterior cingulate (ACC) and dorsolateral prefrontal cortex. Only tasks designed to elicit intentional number processing (a magnitude comparison task) activated a frontoparietal network typically associated with number processing, including the bilateral intraparietal sulci (IPS), the right posterior superior parietal lobule (SPL), and clusters of the frontal cortices. In each of these regions, responses were stronger for item pairs with a smaller numerical distances. A frontoparietal shift was observed with the numeric distance effect, with increased frontal activations and decreased parietal and superior temporal activations with increased GA; however, GA modulated the numeric distance effect only when numeric distance was task-relevant (i.e., during a comparison task but not the perception task).

In a large resting-state fMRI study, Bäuml et al. (2017; *n* = 143) reported that FT and PT participants’ mathematical abilities as children were associated with their full-scale IQ and frontoparietal intrinsic functional connectivity (iFC) as adults, even after controlling for childhood IQ. Right frontoparietal iFC was associated with children’s mathematical abilities and adults’ general cognitive abilities across groups; however, the latter association was significantly stronger in PT participants. Moreover, the authors found differential associations between childhood mathematical abilities and iFC between groups, including a positive correlation between PT participants’ childhood math scores and iFC in the left lateral occipital and middle temporal cortex, while a negative correlation was found among FT participants. Likewise, higher mathematical abilities in childhood were associated with decreased left frontoparietal network connectivity in the superior frontal gyri in PT participants, while this association was reversed for FT participants.

Unique among the PT fMRI studies, Ment et al. (2006; *n* = 71) investigated the impact of a treatment (neonatal administration of indomethacin, a drug that lowers the incidence and severity of intraventricular hemorrhage) on cognitive test scores, achievement in reading and mathematics, and brain activation among a cohort of eight-year-old PT children and FT controls. A previous study involving the same cohort indicated that the protective effects of indomethacin were specific to boys (Ment et al., 2004). The researchers reported that male PT participants assigned to the saline group had lower reading and language scores compared to those assigned to the indomethacin group. They also reported significant treatment-by-gender effects on brain activation in the left inferior parietal lobule, the left IFG (Broca’s area), and the right dorsolateral prefrontal cortex. Activation in these regions was significantly greater in male indomethacin PT participants compared to male PT controls, but not significantly different from that of FT boys, suggesting a protective effect of indomethacin treatment in line with previous research. No such protective effect was reported amongst female participants.

##### Summary

The findings of the functional imaging studies collectively indicate that PT individuals exhibit differences in functional connectivity and neural activation patterns, particularly with respect to language and numeric processing. Amongst these studies, two overarching trends emerged: First, there is evidence for altered right-hemispheric circuit involvement in language processing and reading among PT individuals (Gozzo et al., 2009; Myers et al., 2010). Second, studies focusing on mathematical skills and prematurity suggest atypical activation within the frontoparietal network (Klein et al., 2014; Clark et al., 2017; Klein et al., 2018).

The first trend, increased reliance on the right-hemisphere in language processing, is a deviation from the typical left-lateralization of language functions observed in FT individuals. Altered functional connectivity within this network in PT participants, especially between Wernicke’s area and the right IFG and supramarginal gyri (Gozzo et al., 2009; Myers et al., 2010), indicates a reliance on right-hemispheric networks for language processing. This alternate processing pathway may represent an adaptive response to structural changes or disruptions in typical left-hemispheric language circuits due to prematurity. Alternatively, increased right-hemispheric reliance for language processing in PT youth may reflect maturational delays in the typical developmental trajectory of increasing leftward-lateralization for language (Olulade et al., 2020). These findings align with research indicating that early brain injury may precipitate functional reorganization, allowing other brain regions or networks to compensate for the affected regions (Johnston, 2009).

Concerning numeric skills, Klein et al. (2014, 2018), Bäuml et al. (2017), and Clark et al. (2017), each report atypical frontoparietal network activation in PT individuals. This number processing network appears to mature more slowly in PT individuals, consistently with research demonstrating aberrant frontoparietal functional connectivity in preterm individuals (He and Parikh, 2015). Of note, the functional imaging study by Bäuml et al. (2017) underscores the influence of prematurity on brain-behavior relationships (*cf.* Anderson, 2014), suggesting that mathematical abilities and frontoparietal connectivity are differentially associated in PT and FT individuals.

#### Magnetoencephalography studies

The final prematurity study included in this review used MEG to evaluate differences in cortical activations during a reading phonological task among PT and FT adolescents (Frye et al., 2009; *n* = 31). Participants were subjected to two rhyming tasks (real words and nonsense words) during MEG imaging. During the real-word rhyming task, high-risk PT participants demonstrated a greater mean number of dipole (NOD) moments in the left prefrontal area compared to those in the low-risk PT and FT groups. During the non-word rhyming task, good and average high-risk PT readers demonstrated a greater NOD in the left prefrontal area compared to good and average readers in both the low-risk PT and FT groups. Of note, this pattern was not seen among PT poor readers, suggesting that this prolonged activation may constitute a compensatory mechanism in PT individuals with neonatal complications.

### Alcohol

Prenatal alcohol exposure (PAE) has been linked to a range of neurodevelopmental problems, including fetal alcohol spectrum disorders (FASDs), cognitive and motor impairments, behavioral disorders, learning disorders, intellectual disability, and secondary disabilities (Mattson et al., 2011). The teratogenic effects of alcohol are widespread throughout the brain, affecting both cortical and subcortical structures (Lebel et al., 2011), with the severity of damage correlated to the severity of differences in facial morphology and cognitive function (Nuñez et al., 2011). Children prenatally exposed to alcohol are at increased risk for poor academic achievement (Oei, 2020) and are more likely to require special education (Lupton et al., 2004). According to United States Centers for Disease Control and Prevention (2016), just over 10% of women in the United States have consumed alcohol during pregnancy; moreover, among those aged 35–44, rates of alcohol consumption approach 20%. This is a matter of particular concern, as advanced maternal age (>/=35 years) is an established risk factor for fetal congenital anomalies (Correa-de-Araujo and Yoon, 2021).

Thirteen PAE studies met inclusion criteria. These studies were published between 2008 and 2021, with 61.5% in 2015 or later. Five (38.5%) were conducted in South Africa, followed by Canada (four; 30.8%), and the United States (three; 23.1%), with the remaining study conducted both in the United States and South Africa. Six (46.2%) employed cohort designs, and of those four included comparison participants. Another four (30.8%) employed case–control designs, and three (23.1%) were cross-sectional. Seven (53.9%) had a two-group design, typically with a matched comparison group. One study had a single group, and the remaining five (38.5%) had three or more groups. Finally, five (38.9%) used fMRI; two (15.4%) used dMRI techniques; four (30.8%) used sMRI techniques; one (7.7%) used both sMRI and dMRI techniques; and one (7.7%) EEG. Likewise, five (38.5%) employed a task-based paradigm, and the remaining studies were either resting state (1; 7.7%) or evaluated structural properties of the brain (1; 53.9%).

Sample size was just under 63 (62.85; range 21–139) participants on average. The mean age of participants at the time of the last reported measurement was 12.88, and 52.5% of participants were female. Nearly 70% (69.2%) of PAE studies included data on the race or ethnicity of participants, and 84.6% provided information concerning SES. Achievement constructs examined included mathematics in 53.9% of studies (7), reading and math in 38.5% (5), and reading in 7.7% (1). Standardized achievement measures were used in 69.2% of studies, while the remaining studies employed novel measures (23.1%) or both (7.7%) Of the 12 studies with a control or comparison group, all but one (91.7%) reported decrements in achievement among PAE participants, and all but one reported a significant association between achievement and neuroanatomy or functional activity/connectivity.

The studies generally conformed to quality indicators. All documented participant consent or assent and described participant inclusion criteria in adequate detail, and all included a description of study aims or goals and research hypotheses or exploratory objectives. Study analyses were judged appropriate for all studies; outcome variables were adequately defined; and findings were reported clearly and consistently with the studies’ design. Measures of error or variability were provided for all studies as were obtained probability values. However, four of thirteen (30.8%) failed to report adequate demographic data for participants; two failed to report information on SES (15.4%); and two failed to demonstrate the comparability of groups (15.4%). An overview of study findings, organized by primary imaging modality, is provided below.

#### dMRI studies

Four PAE studies used dMRI to explore the relationships among fetal alcohol exposure, brain development, and achievement. In a longitudinal study, Treit et al. (2013; *n* = 44) examined both volumetric and microstructural properties of the brains of participants with FASD and typically developing controls to better understand the relationships among brain development, academic achievement, executive function, and cognitive abilities. Participants in the FASD group exhibited impaired developmental trajectories of FA and MD in several major tracts between timepoints. Notably, the FASD group showed greater reductions in MD between scans in the SLF, SFOF, and IFOF, which was associated with improved language scores. Additionally, participants with FASD evidenced reduced brain, white, cortical gray, and deep gray matter volumes and smaller age-related increases in brain volume. Moreover, among participants with FASD, decreases in MD in the SLF and SFOF were associated with growth in word reading scores, while no significant association was detected among controls. Likewise, a decrease in SFOF MD was associated with improvements in receptive vocabulary scores, with participants with FASD showing the greatest reductions in MD and having the largest gains in receptive vocabulary scores.

In a small cross-sectional study, the same research team (Lebel et al., 2010; *n* = 21) used DTI to examine the relationship between white-matter properties and math achievement among participants with FASD. A positive correlation was reported between FA and math scores in two clusters in the left parietal lobe and one in the left cerebellum. A negatively correlated cluster was found in the bilateral brainstem. Tractography was used to identify the specific white matter tracts associated with these clusters, which included the left SLF, left CS and CC body, MCP, and bilateral projection fibers (inclusive of the anterior and posterior limbs of the internal capsule).

Finally, Sowell et al. (2008; *n* = 36) also used T_1_-weighted imaging and DTI to examine brain-behavior correlations between a group of participants with FASD and typically-developing matched controls. Analyses revealed significant differences in FA between the FASD and control groups: Specifically, FA was significantly lower in the FASD group in regions of the lateral splenium (medial superior parietal white matter) and posterior cingulate white matter bilaterally, and in the deep white matter of the right temporal lobe. Significant associations between visuomotor integration and FA in bilateral splenium were detected among participants in the FASD group; however, no such correlation was detected among non-exposed participants, and no significant association was found for reading measures and any ROI among participants from either group.

##### Summary

Across the dMRI and PAE studies included in this review, PAE was consistently associated with altered white matter tract development (Sowell et al., 2008; Lebel et al., 2010; Treit et al., 2013). Both Sowell et al. (2008) and Treit et al. (2013) reported widespread reductions in FA across several major fascicles in FASD compared to non-exposed participants. Such a diffuse impact of PAE on white-matter integrity is consistent with a recent systematic review highlighting lower FA and reduced MD/RD in most association and projection fibers, as well as callosal tracts (Ghazi Sherbaf et al., 2019). PAE therefore appears to have detrimental effects throughout the brain, with higher FA and lower MD suggesting lower myelination in FASD, as observed in animal models of PAE (Phillips, 1989; Ozer et al., 2000).

Two studies also reported significant associations between white-matter microstructure and specific aspects of cognitive functioning in individuals with PAE. Treit et al. (2013) found a negative association between measures of MD in the SLF and SFOF and word reading and receptive vocabulary scores, while Lebel et al. (2010) detected positive associations between left parietal and cerebellar FA and math achievement scores. These findings suggest that PAE might have selective, tract-specific effects on cognitive abilities. They also align with the hypothesis that white-matter damage from PAE may lead to less efficient processing and information transfer across the brain, negatively impacting cognitive abilities and neurobehavioral outcomes (Norman et al., 2009).

#### sMRI studies

Volumetric and morphometric analyses represented a common approach to evaluate the relationships among PAE, neurodevelopment, and achievement in reading and mathematics. McLachlan et al. (2020; *n* = 139) focused on the role of SES as a potential moderator of the effects of PAE on regional brain volumes and cognitive abilities amongst children with PAE and non-exposed controls. Performance on study measures, including executive functioning, attention, working memory, math/numerical ability, and word reading, was significantly lower in children with PAE, but SES was not correlated with test scores in either group. PAE was associated with reduced brain volumes (reductions of 4–8%) across all 13 ROIs evaluated, including smaller cerebral and cerebellar grey and white matter volumes and smaller grey matter volume of several subcortical regions. Moreover, higher SES was associated with larger hippocampal and amygdalar volumes in non-exposed children but not in children with PAE, suggesting that the effects of PAE on brain development may not be readily mediated by environmental enrichment.

Gautam et al. (2015, *n* = 139) examined the relationships between longitudinal cortical volume change and arithmetic ability, behavior, and executive function in 7-to-12-year-old children heavily exposed to alcohol before birth and non-exposed peers. Participants received two MRI scans, 2 years apart, and were assessed using standardized evaluations of cognitive abilities, intelligence, behavior, executive functioning, and arithmetic skill. Compared to non-exposed participants, PAE children had lower scores on all neurodevelopmental tests. They also showed reduced white matter and subcortical gray matter volumes. Nevertheless, children in both groups had similar trajectories of brain development, except for growth in the transverse temporal area, which proceeded more slowly among PAE participants. In PAE participants, growth in white matter volume was positively associated with behavioral dysfunction, but negatively correlated among non-exposed peers. Likewise, arithmetic scores were positively associated with growth in the temporal and parietal regions among PAE participants, but no such association was detected among controls.

Miles et al. (2021; *n* = 52) investigated the relationship between brain volumes, cognitive abilities, working memory, and arithmetic skills in a cohort of children with FASDs, heavily-alcohol-exposed non-syndromal children (HE), and non-or minimally-exposed controls. Children in the FASD and HE groups had lower IQ scores, smaller total intracranial volume, and higher blood lead levels than controls. Furthermore, those in the FASD group had reduced volume in the left intraparietal sulcus (IPS), a region implicated in numerical and mathematical processing. Asymmetry in the lateral IPS was evident in the controls but not in those with FASD or HE. Further analyses demonstrated that left and right medial IPS volumes were positively associated with arithmetic skills, but group-related effects were not detected.

In a carefully-designed study, Glass et al. (2017; *n* = 42) performed T_1_-weighted anatomical brain scans on children with PAE and non-exposed controls. Participants with heavy PAE showed deficits in academic performance across domains, including reading, spelling and math, with particularly marked deficits in math functioning. Among controls, surface area in the left superior and inferior parietal lobules, left postcentral gyrus, right middle occipital gyrus, and right middle temporal gyrus was inversely associated with math achievement; this relationship was not detected amongst PAE participants. Likewise, spelling achievement was negatively associated with surface area in bilateral clusters in the inferior and middle temporal gyrus, and fusiform gyrus among controls but not among participants with heavy PAE.

##### Summary

Collectively, the reviewed morphometric and volumetric studies underline the detrimental effects of PAE with substantive evidence of reduced brain volumes and severe cognitive and academic impairments in affected children (Gautam et al., 2015; Glass et al., 2017; McLachlan et al., 2020; Miles et al., 2021). Two notable trends emerged: First, there appears to be a consistent relationship between PAE and reduced cerebral, cerebellar, and subcortical gray matter volumes (Gautam et al., 2015; McLachlan et al., 2020). Second, specific anatomical alterations are associated with lower performance in areas of executive functioning, attention, working memory, and academic abilities, including reading and mathematics (Gautam et al., 2015; Glass et al., 2017; McLachlan et al., 2020; Miles et al., 2021). These findings dovetail with earlier seminal work in the field, including reports of the deleterious effects of FASDs, not only on brain volume but also on the morphological characteristics of specific regions (Archibald et al., 2001), as well as research demonstrating reduced volume of the total brain, cortical, and subcortical structures throughout the lifespan (Rockhold et al., 2023) and upon post-mortem examination (Lebel and Ware, 2023).

Volumetric and morphometric alterations in specific brain regions appear to correlate with the distinct cognitive and academic impairments observed in children PAE. Miles et al. (2021) observed decreased volume of the left IPS, a region linked to numerical and mathematical processing. This reduction aligns with the commonly observed mathematical weakness in PAE children (Rasmussen and Bisanz, 2009). Moreover, Glass et al. (2017) identified correlations between the surface area of various parietal and temporal regions and academic performance, particularly in math and spelling. This relationship was noticeably absent in children with heavy PAE. Gautam et al. (2015) noted that although the general trajectory of brain development was similar in both PAE and control children, growth in certain areas such as the transverse temporal region was slower in PAE participants. This differential pace of development could potentially explain the observed discrepancies in neurodevelopmental outcomes and academic performance.

Of note, both McLachlan et al. (2020) and Miles et al. (2021) reported no significant association between SES and performance on certain neurodevelopmental measures among PAE participants. This might indicate that some of the neurobiological sequelae of PAE are resistant to the mediating effects of environmental enrichment, a noteworthy finding given the significant association between SES and brain development and academic achievement in the general population shown in other research (Hackman and Farah, 2009).

#### fMRI studies

The authors of five PAE studies used fMRI to investigate the relationship between neural activations and performance on evaluations of achievement and cognitive abilities among participants with PAE. As an example, Meintjes et al. (2010; *n* = 33) used functional imaging to explore differences in the neural processes involved in number processing among children with FASDs and typically developing peers. Participants performed two number processing tasks: a proximity judgment task and an exact addition task. Analyses indicated that controls activated the expected frontoparietal network during both tasks, including the anterior and posterior horizontal IPS (with some differences in lateralization between tasks), left precentral sulcus, and posterior medial frontal cortex. During the proximity judgment task, participants with FASDs recruited additional parietal pathways, including the right and left angular gyrus and the posterior cingulate/precuneus, which suggests potential verbal mediation of the task. During the addition task, exposed children showed more diffuse activations, including the cerebellar vermis and cortex, which suggests a lack of fluency and resembles patterns of activation seen among adults working very challenging math problems.

Woods et al. (2018; *n* = 27), examined the neural correlates of non-symbolic numeric processing amongst children with FASDs, heavily-exposed (HE) non-syndromal children, and typically-developing controls from the same community. The IPS was more activated in controls, while HE participants showed enhanced activation in the left angular gyrus with increasing task difficulty. Moreover, in HE children, activation in the right posterior SPL was reduced relative to controls. In a separate group of children, the same research team (Woods et al., 2015; *n* = 49) found a link between PAE and reduced activation in the right anterior horizontal IPS, a region involved in the representation of quantity, during simple addition and number comparison tasks, and reduced posterior SLP activation during a number comparison task. Additionally, the FASD and HE participants demonstrated increased activity in the left angular gyrus during an addition task, suggesting possible compensatory activation. Likewise, Santhanam et al. (2009; *n* = 54) reported that participants with FASD showed significantly lower activation than non-exposed peers in the left superior and right inferior parietal regions and the medial frontal gyrus, during a subtraction task. Non-syndromal HE participants showed similar activation patterns to those seen in participants with FASD, although the difference compared to the control group did not reach statistical significance.

Finally, a single fMRI study used a resting-state paradigm. Little et al. (2018; *n* = 133) acquired fMRI images from participants with FASD and healthy controls, and examined 6 core functional networks. The authors reported similar intranetwork functional connectivity between groups, but lower internetwork functional connectivity in participants with FASD between regions associated with the salience frontoparietal and language networks, reflecting decreased between-network integration.

##### Summary

Collectively, the functional imaging and PAE studies in this review present a consistent pattern of altered activations in children with PAE when performing arithmetic and number processing tasks (Santhanam et al., 2009; Meintjes et al., 2010; Woods et al., 2015, 2018). PAE appears to result in diffuse, widespread activations and recruitment of additional brain regions to perform tasks that normally elicit more localized activation in typically developing controls. Relative over-activation in certain brain regions, such as the angular gyrus (Meintjes et al., 2010; Woods et al., 2015), could be indicative of compensatory cognitive strategies employed by children with FASDs to maintain task performance, a finding consonant with research that has reported differential recruitment of brain regions amongst individuals with FASD on tasks of executive functioning (Diwadkar et al., 2013; Kodali et al., 2017).

By contrast, diminished activation was consistently observed canonical regions for numeric processing, such as the horizontal IPS and posterior SPL (Woods et al., 2015, 2018). This decreased activation in canonical regions responsible for numerical and mathematical cognition aligns with consistent findings of mathematics deficits in children with FASDs (Rasmussen and Bisanz, 2009). Finally, the resting-state fMRI study by Little et al. (2018) points to disrupted functional connectivity as another potential consequence of PAE. This disruption was particularly evident in networks associated with salience, frontoparietal processing, and language, areas often reported to be impaired in individuals with FASDs (Little et al., 2018), cohesive with findings of research linking aberrant connectivity in FASD to cognitive impairments (Wozniak et al., 2017).

#### Electroencephalography studies

Authors of a single study (Ben-Shachar et al., 2020; *n* = 48) used EEG to better understand the relationship between PAE and arithmetic achievement. Adolescents with PAE (non-syndromal HE to FAS) and non-exposed controls were subjected to a simple arithmetic task (distinguishing between correct and incorrect solutions to single-digit addition and subtraction tasks). Participants with PAE had significantly lower accuracy and a weaker neural response to incorrect solutions, reflected by a reduction in low theta-burst activity over frontal regions. Moreover, the severity of PAE was related to the degree of impairment in both the task performance and the brain’s response to errors, consistent with previous research suggesting a potential dose–response effect on FASD severity (Sood et al., 2001).

## Discussion

In the present scoping systematic review, we aimed to identify and synthesize the findings of research in which both brain imaging techniques and assessments of achievement were used to evaluate and understand the relationships among aspects of developmental programming, neurodevelopment, and achievement in reading and mathematics. The results of our review reveal a preponderance of prematurity studies (32), a modest number of PAE studies (13), a single opioid study, and a single organohalogen study. The latter two studies were excluded from the review. Details of these studies can be found in Supplementary Informations S4, S5. Other perinatal insults of interest did not yield search results meeting inclusion criteria. Despite the large number of variables under consideration and the broad heterogeneity in study designs, several salient trends emerged, providing insight into the complex relationship between perinatal insults and neurodevelopmental outcomes. In this section, we discuss trends and limitations and propose directions for future research.

### Prematurity studies

With regard to prematurity, consistent associations were found between poorer white-matter microstructural integrity, particularly lower FA, and poorer cognitive and academic performance among PT participants (Andrews et al., 2010; Thompson et al., 2015; Dubner et al., 2019; Collins et al., 2021). Despite differences in methodologies and brain areas studied, the importance of white-matter integrity in cognitive and academic functioning is clear, consistent with findings from other studies (Qiu et al., 2008; Van Eimeren et al., 2008; Tsang et al., 2009). The critical role of the CC in cognitive functioning and academic performance was underscored across studies reporting significant associations between CC white-matter properties, executive functioning, and achievement in reading and mathematics (Andrews et al., 2010; Thompson et al., 2015; Dubner et al., 2019). These results were extended by Kelly et al. (2016) and Collins et al. (2021), who linked CC maturation rate, fiber density, and fiber-bundle cross-section to mathematics achievement and broader cognitive abilities. Finally, differences in the relationships between white matter properties and cognitive and academic performance between PT and FT participants (Travis et al., 2016; Bruckert et al., 2019; Thompson et al., 2020) indicate altered neurodevelopmental trajectories in PT children, pointing to potentially compensatory neurodevelopmental processes. Such findings may support the hypothesis that neurodevelopmental insults from early life adversity, such as preterm birth, can trigger neural plasticity, leading to the recruitment of alternative neural pathways (Johnson and Marlow, 2017).

PT studies using morphometric and volumetric analyses, moreover, offer evidence of relationships between brain structures and measures of cognitive and academic achievement in PT individuals. Most large-scale studies reported statistically significant associations between brain structure—specifically, volume of white and gray matter and certain brain structures such as the insula, putamen, prefrontal lobes, cerebella, basal ganglia—and measures of academic achievement and cognitive abilities (Allin et al., 2001; Isaacs et al., 2001; Kesler et al., 2006; Scott et al., 2011; Cheong et al., 2013; Ullman et al., 2015; Anderson et al., 2017). Furthermore, several studies detected variations in the relationship between brain morphometry and cognitive/academic outcomes between PT/VPT and FT participants (Allin et al., 2001; Scott et al., 2011; Ullman et al., 2015). These findings are consistent with studies indicating that prematurity alters the trajectory of neurodevelopmental processes (Ment et al., 2009), and neurobehavioral research highlighting the long-term cognitive and academic impact of prematurity (Aarnoudse-Moens et al., 2009). Of particular note, Belfort et al. (2016) found a significant positive association between breastfeeding during the first 28 days of life and better cognitive, linguistic, academic, and motor skills at 7 years of age in VLBW infants. This finding could point to a modifiable factor linked to improved neurodevelopmental outcomes in premature infants.

Finally, the PT functional imaging studies demonstrate a prominent trend of right-hemispheric involvement in reading and language processing among preterm individuals, which is a notable deviation from the typical left-hemispheric activation observed in full-term counterparts (Gozzo et al., 2009; Myers et al., 2010) and parallels observations in poor readers and children with dyslexia (Hoeft et al., 2007). This shift towards the right hemisphere may signify an adaptive neuroplastic response to the structural or developmental alterations caused by premature birth, consistent with research reporting functional reorganization following early brain injury or disrupted development (e.g., Johnston, 2009).

Similarly, fMRI studies examining numerical abilities among preterm individuals provided evidence of atypical activation and slower maturation of the frontoparietal network, a neural circuit frequently associated with numerical processing (Klein et al., 2014, 2018; Bäuml et al., 2017; Clark et al., 2017). These findings are congruent with research showing abnormal frontoparietal functional connectivity in preterm individuals (He and Parikh, 2015). Notably, Bäuml et al. (2017) reported distinctive associations between mathematical abilities and frontoparietal connectivity in PT and FT individuals, suggesting that the neural substrates underlying cognitive functions may be reconfigured by prematurity. This further underscores prematurity as a critical variable influencing brain-behavior relationships (Anderson, 2014).

### Alcohol

Trends among PAE studies were even more pronounced and consistent. Among the studies that used dMRI to explore the impacts of PAE on brain microstructure, several themes emerged. Studies provided converging evidence of an association between PAE and widespread alterations in white matter microstructure (Sowell et al., 2008; Lebel et al., 2010; Treit et al., 2013), specifically reductions in FA across several major fascicles, including the SLF, IFOF, and SFOF (Lebel et al., 2010; Treit et al., 2013). These findings align well with previous literature, supporting the view that pervasive impairment in white matter integrity is a consistent finding among individuals with PAE (Ma et al., 2005; Ghazi Sherbaf et al., 2019), as well as animal-model research indicating that PAE has nocive effects on white matter integrity throughout the brain (Phillips, 1989; Ozer et al., 2000).

Additionally, these microstructural changes were linked with cognitive functioning, underscoring the functional implications of PAE-related brain alterations. Specifically, decreases in MD in the SLF and SFOF were associated with improvements in word reading and receptive vocabulary scores (Treit et al., 2013), while FA values in the left parietal region and cerebellum were positively correlated with math achievement (Lebel et al., 2010). Notably, such tract-specific cognitive associations were observed primarily in individuals with FASD, but less so among controls. This points to a distinctive neurocognitive profile for individuals with PAE, characterized by compromised white matter integrity and associated cognitive deficits.

The PAE morphometric and volumetric studies reviewed provided further evidence of the adverse impacts of PAE on both brain structure and cognitive performance in affected children (Gautam et al., 2015; Glass et al., 2017; McLachlan et al., 2020; Miles et al., 2021). Notably, these studies revealed consistent reductions in cerebral, cerebellar, and subcortical gray matter volumes (Gautam et al., 2015; McLachlan et al., 2020). Moreover, these anatomical alterations were linked to decreased scores on measures of executive functioning, attention, working memory, and academic performance (Gautam et al., 2015; Glass et al., 2017; McLachlan et al., 2020; Miles et al., 2021). These trends align well with earlier research reporting similar neurostructural and neurobehavioral consequences of PAE (Archibald et al., 2001). Likewise, other PAE studies provide evidence of regional specificity of these structural alterations, linking them to characteristic cognitive deficits among affected children. Miles et al. (2021) reported that reductions in the volume of the left IPS, a region implicated in numerical and mathematical processing, corresponded to deficits in arithmetic skills commonly observed in children with PAE. Such regional specificity has also been observed by Glass et al. (2017), who identified distinct relationships between the surface area of various parietal and temporal regions and academic performance in controls, relationships that were conspicuously absent in children with heavy PAE. Of note, Gautam et al. (2015) reported comparable trajectories of brain development between children with PAE and controls, with one notable exception: growth in the transverse temporal area, which was significantly slower in children with PAE. This deviation in growth rate suggests that certain brain regions may be particularly susceptible to the effects of PAE. Finally, while higher SES has been associated with improved brain development and academic achievement in the general population (Hackman and Farah, 2009), the effects of PAE appear to be resistant to such environmental enrichment (McLachlan et al., 2020; Miles et al., 2021). This underscores the severity and persistence of PAE’s effects, highlighting the importance of primary prevention efforts to reduce PAE.

Finally, the PAE fMRI studies revealed consistently altered task activations among children exposed to alcohol *in utero* (Santhanam et al., 2009; Meintjes et al., 2010; Woods et al., 2015, 2018). During number processing tasks, children with PAE exhibited broad, diffused activation, with an over-recruitment of brain regions not typically involved in numeric processing and under-activations in canonical regions for mathematical cognition such as the horizontal IPS or posterior SPL (Woods et al., 2015, 2018). These differences may provide a neural basis for the mathematical deficits consistently observed in children with FASDs (Rasmussen and Bisanz, 2009). Of particular note is the hyperactivation of the angular gyrus, observed by Meintjes et al. (2010) and Woods et al. (2015), which may reveal an over-reliance on language-based arithmetical facts (Dehaene et al., 2003) as an adaptive strategy to compensate for inefficient number processing in core numerical regions (Dehaene et al., 2003), such as the hypo-activated horizontal IPS, a finding consonant with research that has reported differential recruitment of brain regions amongst individuals with FASD on tasks of executive functioning (Diwadkar et al., 2013; Kodali et al., 2017).

Using resting-state fMRI, Little et al. (2018) reported that the adverse effects of PAE extend beyond task-dependent neural responses, implicating intrinsic connectivity of the brain’s functional networks. Specifically, disruptions were observed in networks associated with salience, frontoparietal processing, and language (Little et al., 2018), domains frequently reported as impaired among individuals with FASDs. The observed relationship between disrupted functional connectivity and impaired performance in mathematical reasoning and attention tasks adds to the growing body of research linking aberrant brain connectivity in PAE to cognitive and academic deficits (Wozniak et al., 2017).

## Implications and future directions

Taken together, the findings of the studies included in this review contribute to the broader understanding of the pervasive impacts of perinatal insults, notably prematurity and PAE, on neurodevelopmental outcomes. Despite the heterogeneity in study designs and methodologies, converging evidence underscores the profound influence of perinatal adversity on developmental programming and highlights the urgent need for both primary prevention efforts and timely interventions to mitigate the impacts of these perinatal insults on cognitive and academic performance.

There is an evident dearth of neuroimaging literature on perinatal insults other than prematurity and prenatal alcohol exposure. Specific areas that require further research include opioid and organohalogen exposure, and other under-researched insults, such as maternal hypertensive disease, maternal diabetes, and prenatal exposure to nicotine and heavy metals, in particular organometallic compounds. Given the paucity of studies in these areas, large-scale, multidisciplinary research projects should be initiated. Moreover, there is a clear need for longitudinal studies that follow the neurodevelopmental trajectories from birth to adulthood. This could provide insight into the longer-term effects of these exposures, and the potential for recovery and compensation over time. Such research should focus on establishing links among biological mechanisms, imaging results, and cognitive and behavioral outcomes across a variety of high-prevalence insults. Comparative studies could elucidate insult-specific and shared pathways of neurodevelopmental alteration. Also, more sophisticated epidemiological designs, including sibling-matched studies could provide a more nuanced understanding of how the timing and intensity of these insults interact with genetic and environmental factors to influence neurodevelopmental outcomes.

An increased focus on protective factors and interventions that may mitigate the impacts of perinatal insults would likewise be beneficial. Belfort et al. (2016) noted the potential of breastfeeding as a protective factor in preterm infants, and Ment et al. (2006) explored the neuroprotective effects of indomethacin administration in PT infants. Investigating other potentially modifiable factors, such as maternal health care, nutrition, postnatal environments, or therapeutic intervention could reveal additional means by which to improve neurodevelopmental outcomes.

Though outside of the scope of this review, there is evidence to suggest that infants born PT may experience accelerated maturation of white matter in specific brain regions (Giménez et al., 2008) and ‘catch-up’ growth in head circumference in the extra-uterine environment (Lepomäki et al., 2013)^1^. Given these findings, research should endeavor to explore the relationship, if any, between accelerated or compensatory development in neural parameters and neuro-developmental outcomes in PT individuals.

Finally, there is a need to better integrate diverse imaging modalities. Studies incorporating multiple modalities such as dMRI and functional imaging can offer a more comprehensive understanding of the impacts of perinatal insults on the brain and cognition. Additionally, the use of advanced analytic techniques, including machine learning algorithms and connectivity analyses, could improve the identification of neurodevelopmental patterns unique to each type of insult. This could facilitate early detection and intervention strategies, potentially mitigating the impacts of early-life adversities.

### Limitations of the reviewed studies

While the studies included in this review were generally judged to be of sound quality, the research base was relatively thin, and few studies included repeated measurements. Moreover, many lacked the demographic data necessary to establish the comparability of groups and confirm the absence of demographic confounds. Likewise, several studies had very small sample sizes, and larger cohort studies often had a considerable imbalance between groups, with control groups sometimes having fewer than 15 participants, limiting confidence in the comparisons and the generalizability of results. Finally, there was a great deal of variability in the quality and diversity of study measures, the methods by which effects were detected, how significance was determined, and if/how effect size was calculated. Future research should aim to expand the knowledge base while striving to address the limitations of extant research. Some specific recommendations to address these limitations in future research include:

1. Consistently control for demographic factors—including age, sex, socioeconomic status, and family history—that could influence the relationships between developmental programming and achievement in reading and mathematics.
2. Conduct larger studies with better-balanced groups to increase the statistical power and generalizability of the findings.
3. Replicate existing studies, ideally at a larger scale, in particular those whose findings are unique or represent outliers in their fields.
4. Include a range of cognitive, academic, and behavioral assessments to clarify the domains most affected by perinatal insults.
5. Include repeated measurements to better understand the effects perinatal insults and their causal dynamics, combined with mediation analyses, to clarify the mechanisms and interactions underlying such brain-behavior associations.
6. Collect and report demographic data, in particular race, ethnicity, and SES, to establish the comparability of groups and better control for potential demographic confounds.
7. When possible, ensure that those collecting and analyzing study data are blind to conditions.

### Limitations of the present study

While we aimed to provide a comprehensive overview of the neuroimaging literature on developmental programming and its relationship to reading and mathematics achievement, several limitations should be considered when interpreting the present study’s findings:

1. This review is limited to the examination of a limited number of high-incidence factors affecting developmental programming; numerous other factors affecting developmental programming have been left unexplored.
2. Our research returned literature relevant to the insults of PAE and PT. Prematurity encompasses a spectrum of etiologies including maternal, environmental, and genetic factors, each uniquely influencing neurodevelopment. PAE, by contrast, results from exposure to a teratogen, with more direct and well-defined neurodevelopmental consequences, rendering comparison difficult.
3. The categorization of included articles by (apparent) proximate cause may not reflect the true relationships among the factors.
4. The decision to focus on reading and mathematics achievement may have excluded studies that examined other important neurodevelopmental outcomes or disorders.
5. The decision to examine only English-language articles published in (inter)nationally-circulated, refereed academic journals limited the overall comprehensiveness of the review.
6. Study designs and measures of neuroanatomy, neurofunction, and achievement varied considerably throughout studies, thereby limiting the generalizability of the findings.
7. Inter-rater reliability data for the full-text screening stage were determined by dividing agreements by agreements plus disagreements, which may not reflect the level of agreement attributable to chance.
8. Finally, we did not perform reference chasing, which may have allowed us to identify additional studies meeting inclusion criteria.

## Data availability statement

The original contributions presented in the study are included in the article/Supplementary material, further inquiries can be directed to the corresponding author/s.

## Author contributions

DS: Conceptualization, Investigation, Writing – original draft, Writing – review & editing. FB: Writing – original draft, Writing – review & editing. CR: Writing – original draft, Writing – review & editing. RC: Writing – review & editing. CC: Writing – review & editing. KK: Writing – review & editing. VS: Writing – review & editing. IM: Writing – review & editing. FH: Writing – review & editing.

## Footnotes

^1^However, more rapid growth in head circumference is associated with poorer white-matter integrity per diffusion imaging measures (*Ibid.*).

## Glossary

ACC: anterior cingulate cortex
AD: axial diffusivity
PAE: prenatal alcohol exposure
aSLF: anterior superior longitudinal fasciculus
AF: arcuate fasciculus
CC: corpus callosum
CS: cortico-spinal
CST: corticospinal tract
CSF: cerebrospinal fluid
DTI: diffusion tensor imaging
dMRI: diffusion MRI techniques
DWI: diffusion-weighted imaging
EEG: electroencephalography
EF: executive functioning
FA: fractional anisotropy
FAS: fetal alcohol syndrome
FASD: fetal alcohol spectrum disorders
fMRI: functional magnetic resonance imaging
FOF: frontal-occipital fasciculus
GA: gestational age
HE: heavily alcohol-exposed non-syndromal
I/M/S CP: inferior/middle/superior cerebellar peduncle
ICP: inferior cerebellar peduncle
iFC: intrinsic functional connectivity
IFG: inferior frontal gyrus
IFOF: inferior frontal occipital fasciculus
ILF: inferior longitudinal fasciculus
IQ: intelligence quotient
LBW: low birth weight
MD: mean diffusivity
MEG: magnetoencephalography
MCP: middle cerebellar peduncles
MRI: magnetic resonance imaging
NOD: number of dipole
PCE: prenatal cocaine exposure/exposed
PBDE: polybrominated diphenyl ether
PCB: polychlorinated biphenyl
RD: radial diffusivity
ROI: region of interest
SCP: superior cerebellar peduncles
SES: socioeconomic status
SLF: superior longitudinal fasciculus
S/IFOF: superior/inferior fronto-occipital fasciculus
sMRI: structural MRI techniques
SFOF: superior frontal occipital fasciculus
SPL: superior parietal lobule
TC: thalamo-cortical
VLBW: very/low birth weight
VPT: very preterm

## References

[ref1] Aarnoudse-Moens C. S. Weisglas-Kuperus N. Van Goudoever J. B. Oosterlaan J. (2009). Meta-analysis of neurobehavioral outcomes in very preterm and/or very low birth weight children. Pediatrics 124, 717–728. doi: 10.1542/peds.2008-2816, PMID: 19651588

[ref2] Allin M. Matsumoto H. Santhouse A. M. Nosarti C. Al Asady M. H. Stewart A. L. . (2001). Cognitive and motor function and the size of the cerebellum in adolescents born very pre-term. Brain 124, 60–66. doi: 10.1093/brain/124.1.60, PMID: 11133787

[ref3] American Psychological Association. (2022a). APA PsycArticles. Available at: https://www.apa.org/pubs/databases/psycarticles (Accessed February 8, 2022)

[ref4] American Psychological Association. (2022b). APA PsycInfo. Available at: https://www.apa.org/pubs/databases/psycinfo (Accessed February 8, 2022)

[ref5] Anderson P. J. (2014). Neuropsychological outcomes of children born very preterm. Semin. Fetal Neonatal Med. 19, 90–96. doi: 10.1016/j.siny.2013.11.012, PMID: 24361279

[ref6] Anderson P. J. Treyvaud K. Neil J. J. Cheong J. L. Y. Hunt R. W. Thompson D. K. . (2017). Associations of Newborn brain magnetic resonance imaging with long-term neurodevelopmental impairments in very preterm children. J. Pediatr. 187, 58–65.e1. doi: 10.1016/j.jpeds.2017.04.059, PMID: 28583705 PMC5533625

[ref7] Andrews J. S. Ben-Shachar M. Yeatman J. D. Flom L. L. Luna B. Feldman H. M. (2010). Reading performance correlates with white-matter properties in preterm and term children. Dev. Med. Child Neurol. 52, e94–e100. doi: 10.1111/j.1469-8749.2009.03456.x, PMID: 19747208 PMC2892255

[ref8] Archibald S. L. Fennema-Notestine C. Gamst A. Riley E. P. Mattson S. N. Jernigan T. L. (2001). Brain dysmorphology in individuals with severe prenatal alcohol exposure. Dev. Med. Child Neurol. 43, 148–154. doi: 10.1111/j.1469-8749.2001.tb00179.x, PMID: 11263683

[ref9] Arhan E. Gücüyener K. Soysal Ş. Şalvarlı Ş. Gürses M. A. Serdaroğlu A. . (2017). Regional brain volume reduction and cognitive outcomes in preterm children at low risk at 9 years of age. Child's Nervous Syst. 33, 1317–1326. doi: 10.1007/s00381-017-3421-2, PMID: 28484867

[ref10] Bäuml J. G. Meng C. Daamen M. Baumann N. Busch B. Bartmann P. . (2017). The association of children's mathematic abilities with both adults' cognitive abilities and intrinsic fronto-parietal networks is altered in preterm-born individuals. Brain Struct. Funct. 222, 799–812. doi: 10.1007/s00429-016-1247-4, PMID: 27295131

[ref11] Behnke M. Smith V. C. Committee on Substance Abuse, & Committee on Fetus and Newborn (2013). Prenatal substance abuse: short-and long-term effects on the exposed fetus. Pediatrics 131, e1009–e1024. doi: 10.1542/peds.2012-3931, PMID: 23439891 PMC8194464

[ref12] Belfort M. B. Anderson P. J. Nowak V. A. Lee K. J. Molesworth C. Thompson D. K. . (2016). Breast Milk feeding, brain development, and neurocognitive outcomes: a 7-year longitudinal study in infants Born at less than 30 Weeks' gestation. J. Pediatr. 177, 133–139.e1. doi: 10.1016/j.jpeds.2016.06.045, PMID: 27480198 PMC5037020

[ref13] Ben-Shachar M. S. Shmueli M. Jacobson S. W. Meintjes E. M. Molteno C. D. Jacobson J. L. . (2020). Prenatal alcohol exposure alters error detection during simple arithmetic processing: an electroencephalography study. Alcohol 44, 114–124. doi: 10.1111/acer.14244, PMID: 31742737 PMC6980900

[ref14] Bethlehem R. A. I. Seidlitz J. White S. R. Vogel J. W. Anderson K. M. Adamson C. . (2022). Brain charts for the human lifespan. Nature 604, 525–533. doi: 10.1038/s41586-022-04554-y 35388223 PMC9021021

[ref15] Bijnens E. M. Derom C. Weyers S. Janssen B. G. Thiery E. Nawrot T. S. (2019). Placental mitochondrial DNA content is associated with childhood intelligence. J. Transl. Med. 17:361. doi: 10.1186/s12967-019-2105-y 31703745 PMC6839247

[ref16] Blencowe H. Cousens S. Oestergaard M. Z. Chou D. Moller A. B. Narwal R. . (2012). National, regional, and worldwide estimates of preterm birth rates in the year 2010 with time trends since 1990 for selected countries: a systematic analysis and implications. Lancet 379, 2162–2172. doi: 10.1016/S0140-6736(12)60820-4, PMID: 22682464

[ref17] Bruckert L. Borchers L. R. Dodson C. K. Marchman V. A. Travis K. E. Ben-Shachar M. . (2019). White matter plasticity in Reading-related pathways differs in children born preterm and at term: a longitudinal analysis. Front. Hum. Neurosci. 13:139. doi: 10.3389/fnhum.2019.00139 31139064 PMC6519445

[ref18] Cheong J. L. Anderson P. J. Roberts G. Burnett A. C. Lee K. J. Thompson D. K. . (2013). Contribution of brain size to IQ and educational underperformance in extremely preterm adolescents. PLoS One 8:e77475. doi: 10.1371/journal.pone.0077475, PMID: 24130887 PMC3793949

[ref19] Cherkes-Julkowski M. (1998). Learning disability, attention-deficit disorder, and language impairment as outcomes of prematurity: a longitudinal descriptive study. J. Learn. Disabil. 31, 294–306. doi: 10.1177/002221949803100309, PMID: 9599962

[ref20] Clark C. A. C. Liu Y. Wright N. L. A. Bedrick A. Edgin J. O. (2017). Functional neural bases of numerosity judgments in healthy adults born preterm. Brain Cogn. 118, 90–99. doi: 10.1016/j.bandc.2017.07.011, PMID: 28802184

[ref21] Collins S. E. Spencer-Smith M. Mürner-Lavanchy I. Kelly C. E. Pyman P. Pascoe L. . (2019). White matter microstructure correlates with mathematics but not word reading performance in 13-year-old children born very preterm and full-term. Neuroimage 24:101944. doi: 10.1016/j.nicl.2019.101944, PMID: 31426019 PMC6706654

[ref22] Collins S. E. Thompson D. K. Kelly C. E. Yang J. Y. M. Pascoe L. Inder T. E. . (2021). Development of brain white matter and math computation ability in children born very preterm and full-term. Dev. Cogn. Neurosci. 51:100987. doi: 10.1016/j.dcn.2021.100987, PMID: 34273749 PMC8319459

[ref23] Correa-de-Araujo R. Yoon S. S. S. (2021). Clinical outcomes in high-risk pregnancies due to advanced maternal age. J Womens Health (Larchmt) 30, 160–167. doi: 10.1089/jwh.2020.8860, PMID: 33185505 PMC8020515

[ref24] Dehaene S. Piazza M. Pinel P. Cohen L. (2003). Three parietal circuits for number processing. Cogn. Neuropsychol. 20, 487–506. doi: 10.1080/02643290244000239, PMID: 20957581

[ref25] Diwadkar V. A. Meintjes E. M. Goradia D. Dodge N. C. Warton C. Molteno C. D. . (2013). Differences in cortico-striatal-cerebellar activation during working memory in syndromal and nonsyndromal children with prenatal alcohol exposure. Hum. Brain Mapp. 34, 1931–1945. doi: 10.1002/hbm.22042, PMID: 22451272 PMC6870023

[ref26] Dubner S. E. Dodson C. K. Marchman V. A. Ben-Shachar M. Feldman H. M. Travis K. E. (2019). White matter microstructure and cognitive outcomes in relation to neonatal inflammation in 6-year-old children born preterm. Neuroimage 23:101832. doi: 10.1016/j.nicl.2019.101832, PMID: 31075555 PMC6603335

[ref27] EBSCO Publishing. (2022). Academic search premier. Available at: https://www.ebsco.com/products/research-databases/academic-search-premier (Accessed February 8, 2022)

[ref28] Elsevier. (2022a) Embase. Available at: https://www.embase.com (Accessed February 8, 2022)

[ref29] Elsevier. (2022b). Scopus. Available at: https://www.scopus.com (Accessed February 8, 2024)

[ref30] Feldman H. M. Lee E. S. Yeatman J. D. Yeom K. W. (2012). Language and reading skills in school-aged children and adolescents born preterm are associated with white matter properties on diffusion tensor imaging. Neuropsychologia 50, 3348–3362. doi: 10.1016/j.neuropsychologia.2012.10.014, PMID: 23088817 PMC3631607

[ref31] Frye R. E. Hasan K. Malmberg B. Desouza L. Swank P. Smith K. . (2010). Superior longitudinal fasciculus and cognitive dysfunction in adolescents born preterm and at term. Dev. Med. Child Neurol. 52, 760–766. doi: 10.1111/j.1469-8749.2010.03633.x, PMID: 20187879 PMC2910222

[ref32] Frye R. E. Malmberg B. Desouza L. Swank P. Smith K. Landry S. (2009). Increased prefrontal activation in adolescents born prematurely at high risk during a reading task. Brain Res. 1303, 111–119. doi: 10.1016/j.brainres.2009.09.091, PMID: 19796631 PMC2783693

[ref33] Gautam P. Lebel C. Narr K. L. Mattson S. N. May P. A. Adnams C. M. . (2015). Volume changes and brain-behavior relationships in white matter and subcortical gray matter in children with prenatal alcohol exposure. Hum. Brain Mapp. 36, 2318–2329. doi: 10.1002/hbm.22772, PMID: 25711175 PMC4631525

[ref34] Ghazi Sherbaf F. Aarabi M. H. Hosein Yazdi M. Haghshomar M. (2019). White matter microstructure in fetal alcohol spectrum disorders: a systematic review of diffusion tensor imaging studies. Hum. Brain Mapp. 40, 1017–1036. doi: 10.1002/hbm.24409, PMID: 30289588 PMC6865781

[ref35] Giménez M. Miranda M. J. Born A. P. Nagy Z. Rostrup E. Jernigan T. L. (2008). Accelerated cerebral white matter development in preterm infants: a voxel-based morphometry study with diffusion tensor MR imaging. NeuroImage 41, 728–734. doi: 10.1016/j.neuroimage.2008.02.029, PMID: 18430590

[ref36] Glass L. Moore E. M. Akshoomoff N. Jones K. L. Riley E. P. Mattson S. N. (2017). Academic difficulties in children with prenatal alcohol exposure: presence, profile, and neural correlates. Alcohol 41, 1024–1034. doi: 10.1111/acer.13366, PMID: 28340498 PMC5404947

[ref37] Gozzo Y. Vohr B. Lacadie C. Hampson M. Katz K. H. Maller-Kesselman J. . (2009). Alterations in neural connectivity in preterm children at school age. NeuroImage 48, 458–463. doi: 10.1016/j.neuroimage.2009.06.046, PMID: 19560547 PMC2775072

[ref38] Gregory M. D. Kippenhan J. S. Dickinson D. Carrasco J. Mattay V. S. Weinberger D. R. . (2016). Regional variations in brain gyrification are associated with general cognitive ability in humans. Curr. Biol. 26, 1301–1305. doi: 10.1016/j.cub.2016.03.021, PMID: 27133866 PMC4879055

[ref39] Hackman D. A. Farah M. J. (2009). Socioeconomic status and the developing brain. Trends Cogn. Sci. 13, 65–73. doi: 10.1016/j.tics.2008.11.003, PMID: 19135405 PMC3575682

[ref40] Harrison M. S. Goldenberg R. L. (2016). Global burden of prematurity. Semin. Fetal Neonatal Med. 21, 74–79. doi: 10.1016/j.siny.2015.12.007, PMID: 26740166

[ref41] He L. Parikh N. A. (2015). Aberrant executive and Frontoparietal functional connectivity in very preterm infants with diffuse White matter abnormalities. Pediatr. Neurol. 53, 330–337. doi: 10.1016/j.pediatrneurol.2015.05.001, PMID: 26216502

[ref42] Higgins J. P. T. Thomas J. Chandler J. Cumpston M. Li T. Page M. . (2019). Cochrane handbook for systematic reviews of interventions. Wiley-Blackwell. https://training.cochrane.org/handbook/current 10.1002/14651858.ED000142 PMC10284251 31643080

[ref43] Hoeft F. Meyler A. Hernandez A. Juel C. Taylor-Hill H. Martindale J. L. . (2007). Functional and morphometric brain dissociation between dyslexia and reading ability. Proc. Natl. Acad. Sci. U. S. A. 104, 4234–4239. doi: 10.1073/pnas.0609399104, PMID: 17360506 PMC1820738

[ref44] Institute of Education Sciences. (2022). ERIC. Available at: https://eric.ed.gov (Accessed February 8, 2022)

[ref45] Irner T. B. (2012). Substance exposure in utero and developmental consequences in adolescence: a systematic review. Child Neuropsychol. 18, 521–549. doi: 10.1080/09297049.2011.628309, PMID: 22114955

[ref46] Isaacs E. B. Edmonds C. J. Lucas A. Gadian D. G. (2001). Calculation difficulties in children of very low birthweight: a neural correlate. Brain 124, 1701–1707. doi: 10.1093/brain/124.9.1701, PMID: 11522573

[ref47] Johnson S. Marlow N. (2017). Early and long-term outcome of infants born extremely preterm. Arch. Dis. Child. 102, 97–102. doi: 10.1136/archdischild-2015-309581 27512082

[ref48] Johnston M. V. (2009). Plasticity in the developing brain: implications for rehabilitation. Dev. Disabil. Res. Rev. 15, 94–101. doi: 10.1002/ddrr.64, PMID: 19489084

[ref49] Kelly C. E. Thompson D. K. Chen J. Leemans A. Adamson C. L. Inder T. E. . (2016). Axon density and axon orientation dispersion in children born preterm. Hum. Brain Mapp. 37, 3080–3102. doi: 10.1002/hbm.23227, PMID: 27133221 PMC5524572

[ref50] Kershner J. R. (2020a). An evolutionary perspective of dyslexia, stress, and brain network homeostasis. Front. Hum. Neurosci. 14:575546. doi: 10.3389/fnhum.2020.575546 33551772 PMC7859477

[ref51] Kershner J. R. (2020b). Dyslexia as an adaptation to cortico-limbic stress system reactivity. Neurobiol. Stress 12:100223. doi: 10.1016/j.ynstr.2020.100223 32435671 PMC7231974

[ref52] Kesler S. R. Vohr B. Schneider K. C. Katz K. H. Makuch R. W. Reiss A. L. . (2006). Increased temporal lobe gyrification in preterm children. Neuropsychologia 44, 445–453. doi: 10.1016/j.neuropsychologia.2005.05.015, PMID: 15985272

[ref53] Kirkegaard I. Obel C. Hedegaard M. Henriksen T. B. (2006). Gestational age and birth weight in relation to school performance of 10-year-old children: a follow-up study of children born after 32 completed weeks. Pediatrics 118, 1600–1606. doi: 10.1542/peds.2005-2700, PMID: 17015552

[ref54] Klein E. Moeller K. Huber S. Willmes K. Kiechl-Kohlendorfer U. Kaufmann L. (2018). Gestational age modulates neural correlates of intentional, but not automatic number magnitude processing in children born preterm. Int. J. Dev. Neurosci. 65, 38–44. doi: 10.1016/j.ijdevneu.2017.10.004, PMID: 29037913

[ref55] Klein E. Moeller K. Kiechl-Kohlendorfer U. Kremser C. Starke M. Cohen Kadosh R. . (2014). Processing of intentional and automatic number magnitudes in children born prematurely: evidence from fMRI. Dev. Neuropsychol. 39, 342–364. doi: 10.1080/87565641.2014.939179, PMID: 25090014 PMC4270260

[ref56] Kodali V. N. Jacobson J. L. Lindinger N. M. Dodge N. C. Molteno C. D. Meintjes E. M. . (2017). Differential recruitment of brain regions during response inhibition in children prenatally exposed to alcohol. Alcohol 41, 334–344. doi: 10.1111/acer.13307, PMID: 28075019 PMC5272840

[ref57] Lammertink F. Vinkers C. H. Tataranno M. L. Benders M. J. N. L. (2021). Premature birth and developmental programming: mechanisms of resilience and vulnerability. Front. Psych. 11:531571. doi: 10.3389/fpsyt.2020.531571, PMID: 33488409 PMC7820177

[ref58] Landi N. Avery T. Crowley M. J. Wu J. Mayes L. (2017). Prenatal cocaine exposure impacts language and Reading into late adolescence: Behavioral and ERP evidence. Dev. Neuropsychol. 42, 369–386. doi: 10.1080/87565641.2017.1362698, PMID: 28949778 PMC5822684

[ref59] Langley-Evans S. C. (2006). Developmental programming of health and disease. Proc. Nutr. Soc. 65, 97–105. doi: 10.1079/PNS2005478, PMID: 16441949 PMC1885472

[ref60] Lebel C. Rasmussen C. Wyper K. Andrew G. Beaulieu C. (2010). Brain microstructure is related to math ability in children with fetal alcohol spectrum disorder. Alcohol 34, 354–363. doi: 10.1111/j.1530-0277.2009.01097.x, PMID: 19930234

[ref61] Lebel C. Roussotte F. Sowell E. R. (2011). Imaging the impact of prenatal alcohol exposure on the structure of the developing human brain. Neuropsychol. Rev. 21, 102–118. doi: 10.1007/s11065-011-9163-0, PMID: 21369875 PMC3098972

[ref62] Lebel C. Ware A. (2023). “Magnetic resonance imaging in fetal alcohol spectrum disorder (FASD)” in Neurodevelopmental Pediatrics: Genetic and environmental influences. eds. Eisenstat D. D. Goldowitz D. Oberlander T. F. Yager J. Y. (Cham: Springer International Publishing), 397–407.

[ref63] Lee H. J. Park H. K. (2016). Neurodevelopmental outcome of preterm infants at childhood: cognition and language. Hanyang Med. Rev. 36:55. doi: 10.7599/hmr.2016.36.1.55, PMID: 38165408

[ref64] Lepomäki V. Leppänen M. Matomäki J. Lapinleimu H. Lehtonen L. Haataja L. . (2013). Preterm infants' early growth and brain white matter maturation at term age. Pediatr. Radiol. 43, 1357–1364. doi: 10.1007/s00247-013-2699-9, PMID: 23794054

[ref65] Little G. Reynolds J. Beaulieu C. (2018). Altered functional connectivity observed at rest in children and adolescents prenatally exposed to alcohol. Brain Connect. 8, 503–515. doi: 10.1089/brain.2017.0572, PMID: 30289280

[ref66] Loeb D. F. Imgrund C. M. Lee J. Barlow S. M. (2020). Language, motor, and cognitive outcomes of toddlers who were Born preterm. Am. J. Speech Lang. Pathol. 29, 625–637. doi: 10.1044/2019_AJSLP-19-00049, PMID: 32130865 PMC7842870

[ref9001] Lupton C. Burd L. Harwood R. (2004). Cost of fetal alcohol spectrum disorders. Am. J. Med. Genet. 127C, 42–50. doi: 10.1002/ajmg.c.30015 15095471

[ref67] Ma X. Coles C. D. Lynch M. E. LaConte S. M. Zurkiya O. Wang D. . (2005). Evaluation of corpus callosum anisotropy in young adults with fetal alcohol syndrome according to diffusion tensor imaging. Alcohol 29, 1214–1222. doi: 10.1097/01.ALC.0000171934.22755.6D, PMID: 16046877

[ref68] Manuck T. A. Rice M. M. Bailit J. L. Grobman W. A. Reddy U. M. Wapner R. J. . (2016). Preterm neonatal morbidity and mortality by gestational age: a contemporary cohort. Am. J. Obstet. Gynecol. 215, 103.e1–103.e14. doi: 10.1016/j.ajog.2016.01.004, PMID: 26772790 PMC4921282

[ref9002] Margolis A. E. Banker S. Pagliaccio D. De Water E. Curtin P. Bonilla A. . (2020). Functional connectivity of the reading network is associated with prenatal polybrominated diphenyl ether concentrations in a community sample of 5 year-old children: A preliminary study. Environ. Int. 134:105212. doi: 10.1016/j.envint.2019.105212 31743804 PMC7048018

[ref69] Marks G. N. (2006). Influences on, and the consequences of, low achievement. Aust. Educ. Res. 33, 95–115. doi: 10.1007/BF03246283, PMID: 38727342

[ref70] Mascheretti S. Bureau A. Battaglia M. Simone D. Quadrelli E. Croteau J. . (2013). An assessment of gene-by-environment interactions in developmental dyslexia-related phenotypes. Genes Brain Behav. 12, 47–55. doi: 10.1111/gbb.12000, PMID: 23176554

[ref71] Mattson S. N. Crocker N. Nguyen T. T. (2011). Fetal alcohol spectrum disorders: neuropsychological and behavioral features. Neuropsychol. Rev. 21, 81–101. doi: 10.1007/s11065-011-9167-9, PMID: 21503685 PMC3410672

[ref72] McLachlan K. Zhou D. Little G. Rasmussen C. Pei J. Andrew G. . (2020). Current socioeconomic status correlates with brain volumes in healthy children and adolescents but not in children with prenatal alcohol exposure. Front. Hum. Neurosci. 14:223. doi: 10.3389/fnhum.2020.00223 32714166 PMC7344164

[ref73] Meintjes E. M. Jacobson J. L. Molteno C. D. Gatenby J. C. Warton C. Cannistraci C. J. . (2010). An FMRI study of number processing in children with fetal alcohol syndrome. Alcohol 34, 1450–1464. doi: 10.1111/j.1530-0277.2010.01230.x, PMID: 20528824

[ref74] Ment L. R. Kesler S. Vohr B. Katz K. H. Baumgartner H. Schneider K. C. . (2009). Longitudinal brain volume changes in preterm and term control subjects during late childhood and adolescence. Pediatrics 123, 503–511. doi: 10.1542/peds.2008-0025, PMID: 19171615 PMC2679898

[ref75] Ment L. R. Peterson B. S. Meltzer J. A. Vohr B. Allan W. Katz K. H. . (2006). A functional magnetic resonance imaging study of the long-term influences of early indomethacin exposure on language processing in the brains of prematurely born children. Pediatrics 118, 961–970. doi: 10.1542/peds.2005-2870, PMID: 16950986 PMC2364718

[ref76] Ment L. R. Vohr B. R. Makuch R. W. Westerveld M. Katz K. H. Schneider K. C. . (2004). Prevention of intraventricular hemorrhage by indomethacin in male preterm infants. J. Pediatr. 145, 832–834. doi: 10.1016/j.jpeds.2004.07.035, PMID: 15580211

[ref77] Miles M. Warton F. L. Meintjes E. M. Molteno C. D. Jacobson J. L. Jacobson S. W. . (2021). Effects of prenatal alcohol exposure on the volumes of the lateral and medial walls of the intraparietal sulcus. Front. Neuroanat. 15:639800. doi: 10.3389/fnana.2021.639800, PMID: 34163333 PMC8215540

[ref78] Myers E. H. Hampson M. Vohr B. Lacadie C. Frost S. J. Pugh K. R. . (2010). Functional connectivity to a right hemisphere language center in prematurely born adolescents. NeuroImage 51, 1445–1452. doi: 10.1016/j.neuroimage.2010.03.049, PMID: 20347043 PMC2872040

[ref79] National Center for Biotechnology Information. (2022). PubMed. Available at: https://www.ebsco.com/products/research-databases/medline (Accessed February 8, 2022)

[ref80] National Heart, Lung, and blood institute, National Institutes of Health. (2013).Study quality assessment tools. Available at: https://www.nhlbi.nih.gov/health-topics/study-quality-assessment-tools (Accessed February 21, 2023)

[ref81] National Library of Medicine. (2022). MedLine. Available at: http://pubmed.ncbi.nlm.nih.gov (Accessed February 8, 2022)

[ref82] Norman A. L. Crocker N. Mattson S. N. Riley E. P. (2009). Neuroimaging and fetal alcohol spectrum disorders. Dev. Disabil. Res. Rev. 15, 209–217. doi: 10.1002/ddrr.72, PMID: 19731391 PMC3442778

[ref83] Nuñez C. C. Roussotte F. Sowell E. R. (2011). Focus on: structural and functional brain abnormalities in fetal alcohol spectrum disorders. Alcohol Res. Health 34, 121–131, PMID: 23580049 PMC3860550

[ref84] Oei J. L. (2020). Alcohol use in pregnancy and its impact on the mother and child. Addiction 115, 2148–2163. doi: 10.1111/add.15036, PMID: 32149441

[ref85] Olulade O. A. Seydell-Greenwald A. Chambers C. E. Turkeltaub P. E. Dromerick A. W. Berl M. M. . (2020). The neural basis of language development: changes in lateralization over age. Proc. Natl. Acad. Sci. USA 117, 23477–23483. doi: 10.1073/pnas.1905590117, PMID: 32900940 PMC7519388

[ref86] Ozer E. Sarioglu S. Güre A. (2000). Effects of prenatal ethanol exposure on neuronal migration, neuronogenesis and brain myelination in the mice brain. Clin. Neuropathol. 19, 21–25, PMID: 10774947

[ref87] Page M. J. McKenzie J. E. Bossuyt P. M. Boutron I. Hoffmann T. C. Mulrow C. D. . (2021). The PRISMA 2020 statement: an updated guideline for reporting systematic reviews. BMJ 372:n71. doi: 10.1136/bmj.n71, PMID: 33782057 PMC8005924

[ref88] Paul L. K. (2011). Developmental malformation of the corpus callosum: a review of typical callosal development and examples of developmental disorders with callosal involvement. J. Neurodev. Disord. 3, 3–27. doi: 10.1007/s11689-010-9059-y, PMID: 21484594 PMC3163989

[ref89] Pavlova M. Sokolov A. N. Krägeloh-Mann I. (2009). Arithmetic and brain connectivity: mental calculation in adolescents with periventricular lesions. Neuropsychologia 47, 439–445. doi: 10.1016/j.neuropsychologia.2008.09.014, PMID: 18929585

[ref90] Pérez-Pereira M. Peralbo M. Veleiro A. (2017). “Executive functions and language development in pre-term and full-term children” in Language development and disorders in Spanish-speaking children. eds. Auza Benavides A. Schwartz R. G., vol. 14 (Cham: Springer International Publishing), 91–112.

[ref91] Phillips D. E. (1989). Effects of limited postnatal ethanol exposure on the development of myelin and nerve fibers in rat optic nerve. Exp. Neurol. 103, 90–100. doi: 10.1016/0014-4886(89)90190-8, PMID: 2912754

[ref92] Qiu D. Tan L. H. Zhou K. Khong P. L. (2008). Diffusion tensor imaging of normal white matter maturation from late childhood to young adulthood: voxel-wise evaluation of mean diffusivity, fractional anisotropy, radial and axial diffusivities, and correlation with reading development. NeuroImage 41, 223–232. doi: 10.1016/j.neuroimage.2008.02.023, PMID: 18395471

[ref93] Raffington L. Tanksley P. T. Sabhlok A. Vinnik L. Mallard T. King L. S. . (2021). Socially stratified epigenetic profiles are associated with cognitive functioning in children and adolescents. Psychol. Sci. 34, 170–185. doi: 10.1177/09567976221122760 PMC10068508 36459657

[ref94] Räikkönen K. Seckl J. R. Pesonen A.-K. Simons A. Van den Bergh B. R. H. (2011). Stress, glucocorticoids and liquorice in human pregnancy: programmers of the offspring brain. Stress 14, 590–603. doi: 10.3109/10253890.2011.602147, PMID: 21875300

[ref95] Rasmussen C. Bisanz J. (2009). Exploring mathematics difficulties in children with fetal alcohol spectrum disorders. Child Dev. Perspect. 3, 125–130. doi: 10.1111/j.1750-8606.2009.00091.x, PMID: 28340498

[ref96] Rockhold M. N. Donald K. A. Kautz-Turnbull C. Petrenko C. L. (2023). “Neuroimaging findings in FASD across the lifespan” in Fetal alcohol spectrum disorders: a multidisciplinary approach. eds. Abdul-Rahman O. A. Petrenko C. L. M. (Cham: Springer International Publishing), 187–219.

[ref97] Santhanam P. Li Z. Hu X. Lynch M. E. Coles C. D. (2009). Effects of prenatal alcohol exposure on brain activation during an arithmetic task: an fMRI study. Alcohol 33, 1901–1908. doi: 10.1111/j.1530-0277.2009.01028.x, PMID: 19673738 PMC3796099

[ref98] Schneider-Richardson D. A. Hoeft F. Bouhali F. Richter C. G. (2021). The relationships among perinatal developmental programming, neurodevelopment and achievement in reading and mathematics. OSF.

[ref99] Scott F. E. Mechelli A. Allin M. P. Walshe M. Rifkin L. Murray R. M. . (2011). Very preterm adolescents show gender-dependent alteration of the structural brain correlates of spelling abilities. Neuropsychologia 49, 2685–2693. doi: 10.1016/j.neuropsychologia.2011.05.016, PMID: 21651922

[ref100] Sood B. Delaney-Black V. Covington C. Nordstrom-Klee B. Ager J. Templin T. . (2001). Prenatal alcohol exposure and childhood behavior at age 6 to 7 years: I. Dose-response effect. Pediatrics 108:E34. doi: 10.1542/peds.108.2.e34 11483844

[ref101] Sowell E. R. Johnson A. Kan E. Lu L. H. Van Horn J. D. Toga A. W. . (2008). Mapping white matter integrity and neurobehavioral correlates in children with fetal alcohol spectrum disorders. J. Neurosci. 28, 1313–1319. doi: 10.1523/JNEUROSCI.5067-07.2008, PMID: 18256251 PMC3567846

[ref102] Stewart A. L. Rifkin L. Amess P. N. Kirkbride V. Townsend J. P. Miller D. H. . (1999). Brain structure and neurocognitive and behavioural function in adolescents who were born very preterm. Lancet 353, 1653–1657. doi: 10.1016/S0140-6736(98)07130-X, PMID: 10335784

[ref103] Thompson D. K. Lee K. J. Van Bijnen L. Leemans A. Pascoe L. Scratch S. E. . (2015). Accelerated corpus callosum development in prematurity predicts improved outcome. Hum. Brain Mapp. 36, 3733–3748. doi: 10.1002/hbm.22874, PMID: 26108187 PMC6868949

[ref104] Thompson D. K. Loh W. Y. Connelly A. Cheong J. L. Y. Spittle A. J. Chen J. . (2020). Basal ganglia and thalamic tract connectivity in very preterm and full-term children; associations with 7-year neurodevelopment. Pediatr. Res. 87, 48–56. doi: 10.1038/s41390-019-0546-x, PMID: 31486778

[ref105] Travis K. E. Ben-Shachar M. Myall N. J. Feldman H. M. (2016). Variations in the neurobiology of reading in children and adolescents born full term and preterm. Neuroimage 11, 555–565. doi: 10.1016/j.nicl.2016.04.003, PMID: 27158588 PMC4845391

[ref106] Travis K. E. Leitner Y. Feldman H. M. Ben-Shachar M. (2015). Cerebellar white matter pathways are associated with reading skills in children and adolescents. Hum. Brain Mapp. 36, 1536–1553. doi: 10.1002/hbm.22721, PMID: 25504986 PMC4374012

[ref107] Treit S. Lebel C. Baugh L. Rasmussen C. Andrew G. Beaulieu C. (2013). Longitudinal MRI reveals altered trajectory of brain development during childhood and adolescence in fetal alcohol spectrum disorders. J. Neurosci. 33, 10098–10109. doi: 10.1523/JNEUROSCI.5004-12.2013, PMID: 23761905 PMC6618394

[ref108] Tricco A. C. Lillie E. Zarin W. O'Brien K. K. Colquhoun H. Levac D. . (2018). PRISMA extension for scoping reviews (PRISMA-ScR): checklist and explanation. Ann. Intern. Med. 169, 467–473. doi: 10.7326/M18-0850, PMID: 30178033

[ref109] Tsang J. M. Dougherty R. F. Deutsch G. K. Wandell B. A. Ben-Shachar M. (2009). Frontoparietal white matter diffusion properties predict mental arithmetic skills in children. Proc. Natl. Acad. Sci. U. S. A. 106, 22546–22551. doi: 10.1073/pnas.0906094106, PMID: 19948963 PMC2799736

[ref110] Ullman H. Spencer-Smith M. Thompson D. K. Doyle L. W. Inder T. E. Anderson P. J. . (2015). Neonatal MRI is associated with future cognition and academic achievement in preterm children. Brain 138, 3251–3262. doi: 10.1093/brain/awv244, PMID: 26329284 PMC4731414

[ref111] United States Centers for Disease Control and Prevention. (2016). CDC press release on alcohol consumption during pregnancy. CDC. Available at: https://www.cdc.gov/media/releases/2015/p0924-pregnant-alcohol.html

[ref112] Van Eimeren L. Niogi S. N. McCandliss B. D. Holloway I. D. Ansari D. (2008). White matter microstructures underlying mathematical abilities in children. Neuroreport 19, 1117–1121. doi: 10.1097/WNR.0b013e328307f5c1, PMID: 18596611

[ref113] Van Ettinger-Veenstra H. Widén C. Engström M. Karlsson T. Leijon I. Nelson N. (2017). Neuroimaging of decoding and language comprehension in young very low birth weight (VLBW) adolescents: indications for compensatory mechanisms. PLoS One 12:e0185571. doi: 10.1371/journal.pone.0185571, PMID: 28968426 PMC5624616

[ref114] Van Handel M. Swaab H. de Vries L. S. Jongmans M. J. (2007). Long-term cognitive and behavioral consequences of neonatal encephalopathy following perinatal asphyxia: a review. Eur. J. Pediatr. 166, 645–654. doi: 10.1007/s00431-007-0437-8, PMID: 17426984 PMC1914268

[ref115] Veritas Health Innovation. (2022). Covidence systematic review software. Melbourne, Australia. Available at www.covidence.org

[ref116] Viteri O. A. Soto E. E. Bahado-Singh R. O. Christensen C. W. Chauhan S. P. Sibai B. M. (2015). Fetal anomalies and long-term effects associated with substance abuse in pregnancy: a literature review. Am. J. Perinatol. 32, 405–416. doi: 10.1055/s-0034-1393932, PMID: 25486291

[ref117] Williams J. H. G. Ross L. (2007). Consequences of prenatal toxin exposure for mental health in children and adolescents: a systematic review. Eur. Child Adolesc. Psychiatry 16, 243–253. doi: 10.1007/s00787-006-0596-6, PMID: 17200791

[ref118] Woods K. J. Jacobson S. W. Molteno C. D. Jacobson J. L. Meintjes E. M. (2018). Altered parietal activation during non-symbolic number comparison in children with prenatal alcohol exposure. Front. Hum. Neurosci. 11:627. doi: 10.3389/fnhum.2017.00627 29358911 PMC5766638

[ref119] Woods K. J. Meintjes E. M. Molteno C. D. Jacobson S. W. Jacobson J. L. (2015). Parietal dysfunction during number processing in children with fetal alcohol spectrum disorders. Neuroimage 8, 594–605. doi: 10.1016/j.nicl.2015.03.023, PMID: 26199871 PMC4506983

[ref120] Wozniak J. R. Mueller B. A. Mattson S. N. Coles C. D. Kable J. A. Jones K. L. . (2017). Functional connectivity abnormalities and associated cognitive deficits in fetal alcohol Spectrum disorders (FASD). Brain Imaging Behav. 11, 1432–1445. doi: 10.1007/s11682-016-9624-4, PMID: 27734306 PMC5389933

[ref121] Yeoh S. L. Eastwood J. Wright I. M. Morton R. Melhuish E. Ward M. . (2019). Cognitive and motor outcomes of children with prenatal opioid exposure: a systematic review and meta-analysis. JAMA Netw. Open 2:e197025. doi: 10.1001/jamanetworkopen.2019.7025, PMID: 31298718 PMC6628595

