# Supplementary material for: Perinatal influences on academic achievement and the developing brain: a scoping systematic review
Source: Front Psychol. 2024 Jun 19;15:1352241. doi: 10.3389/fpsyg.2024.1352241 (PMC11221367; doi:10.3389/fpsyg.2024.1352241)
Supplement: Supplementary file 1 [file Data_Sheet_1.docx]

**Supporting Information**

**Table S1**

*Detailed search terms*

| **Group A** | arith* OR decod* OR dyscalcul* OR dyslexi* OR “language arts” OR literacy OR read* OR math* OR phonem* OR phonetic OR phonics OR phonologic* OR “ELA” |
| --- | --- |
| **Group B** | “brain imag*” OR “brain-imag*” OR "diffusion imag*" OR "diffusion MRI" OR "diffusion tensor" OR "diffusion-tensor" OR "diffusion weighted" OR "diffusion-weighted" OR electroencephalogra* OR “electro-encephalogra*” OR "event related" OR "event-related" OR "functional MRI" OR “magnetic resonance*” OR "magnetic resonance imag*" OR "magnetic-resonance imag*" OR "magnetic resonance spectroscop*" OR "magnetic-resonance spectroscop*" OR magnetoencephalogra* OR “magneto-encephalogra*” OR neuroimag* OR “neuro-imag*” OR "positron emission" OR "positron-emission" OR “single photon emission” OR "single-photon emission" OR DCS OR DTI OR DWI OR “EEG” OR “ERP” OR fMRI OR “MEG” OR MRI OR MRS OR “PET” OR SPECT OR tDCS OR TMS |
| **Group C** | addict* OR alcohol* OR asphyx* OR cannabis OR cigarette* OR cocaine OR “drug abuse” OR “drug use” OR eclampsia OR “elevated blood pressure” OR “halogenated organic” OR “heavy metal” OR “high blood pressure” OR hyperbilirubin* OR “hyper-bilirubin*” OR hypertens* OR “hyper-tens*” OR hypox* OR “illegal drug*” OR “illicit drug*” OR infect* OR jaundice* OR lead OR marijuana OR mercury OR methylmercury OR “methyl-mercury” OR nicotin* OR organohalogen* OR “organo-halogen*” OR opiate* OR opioid* OR plumbism OR polybrominated* OR "poly-brominated*" OR polychlorinated* OR "poly-chlorinated*" OR preeclampsia OR "pre-eclampsia" OR prematur* OR “pre-matur*” OR preterm OR “pre-term” OR sepsis OR septicemia OR smok* OR “substance abuse” OR “substance use” OR toxemia OR tobacco OR “FAS” OR FASD OR FASDs OR PBDE OR PBDEs OR PCB OR PCBs |
| **Group D** | antepartum OR “ante-partum” OR antenatal OR “ante-natal” OR babies OR baby OR birth* OR *born* OR fetal OR fetus* OR foetal OR foetus* OR infant* OR intrapartum OR “intra-partum” OR matern* OR mother* OR neonat* OR “neo-nat*” OR newborn OR “new-born” OR perinatal OR “peri-natal” OR pregnan* OR prenatal OR “pre-natal” OR prematur* OR “pre-matur*” OR preterm OR “pre-term” OR “post natal” OR “postnatal” OR “post-natal” |

Note: Terms from groups A, B, C, and D were combined using the operator AND.

## **S1. Screening process**

## The first stage of the review process, title and abstract screening, was conducted independently for each article by at least two trained study personnel. Inter-rater reliability at this stage was determined by dividing the total number of agreements by the total number of agreements plus disagreements. After initial title and abstract screening, articles containing keywords in conformity with the selection criteria were retained for full-text screening. Disagreements at this stage were resolved by the study’s first author. The second stage, full-text screening, was again conducted independently for each article by at least two trained study personnel. Inter-rater reliability was again determined by dividing the total number of agreements by the total number of agreements plus disagreements. Disagreements at this stage were again resolved by the study’s first author. In the third stage, extraction and coding, data derived from studies retained at the full-text screening stage were extracted and coded by study personnel. Given the complexity of the information extracted, data extraction was completed by the first author in tandem with her undergraduate research assistants, arriving at mutually agreed upon codes through an iterative process of dialogue and consensus. Consequently, inter-rater reliability data are not available for data extraction.

**S2. Evaluation of study quality**

Each item was evaluated using a true/false, forced-choice response.

1. Are the study's aims and/or goals clearly delineated?
2. Do the study's authors provide a clearly delineated research hypothesis or a statement of exploratory aims?
3. Do the study's authors clearly describe participant inclusion criteria?
4. Do the study's authors document the receipt of informed consent/assent from participants?
5. Are sufficient demographic data provided for participants, including age, gender, and race/ethnicity?
6. Are SES data reported by the authors?
7. Do the study's authors demonstrate the comparability of groups at baseline, or do they statistically correct for reported differences among groups in their analyses?
8. Are the main outcome variables clearly described in the introduction or methods section?
9. Are the study's findings clearly reported for each outcome?
10. Is outcome data reported completely and consistently with the research design?
11. Have measures of error and/or variability been provided for each outcome where appropriate?
12. Have obtained probability values been reported (eg, 0.035 rather than <0.05) for the main outcomes except where the probability value is <0.001?

**S3. Effect Size**

We elected not to consider effect size as a quality indicator for the studies included in this review, nor did we attempt to calculate the effect size for any effect reported in any study.

1. Diversity of Effects and Regions Evaluated: There were few consistent trends in effects or regions evaluated across studies, rendering generalization difficult, even in cases where effect size was reported.
2. Sparsity of Reported Effect Size: Fewer than ten percent of the studies included in this review reported measures of effect size, and given the diversity of techniques used in study analyses, there was no straightforward means by which to calculate effect size across studies.
3. Lack of Standardization: There is no widely accepted standard for calculating effect sizes in brain imaging research. While several models have been proposed (e.g. Durnez et al., 2016; Mumford, 2012; Roiser et al., 2016), many are limited by study design, imaging technique, or data-analysis strategy, and we are not aware of a model that would allow for a straightforward comparison of the magnitude of effects among the various neuroimaging studies included in this review.
4. Multiple Comparisons: Brain imaging studies routinely involve multiple comparisons, sometimes involving information derived from tens of thousands of voxels and dozens of ROIs; as such typical correction procedures, such as Bonferroni’s correction for multiple comparisons, are often ill-suited to data derived from neuroimaging studies (Cremers, Wager & Yarkoni, 2017; Lindquist & Mejia, 2015).
5. Small Sample Sizes: Brain imaging research often involves relatively small sample sizes, which can limit the power of the study and increase the uncertainty of the results. This may make it more difficult for researchers to detect and report meaningful effect sizes (cf. Button et al., 2013; Turner, Paul, Miller, and Barbey, 2018).

**S4. Opioids**

Opioid exposure constitutes an important and increasing threat to perinatal developmental programming. According to the United States Centers for Disease Control and Prevention (2020), about 7% of women reported the use of prescription opioid drugs during pregnancy, and research indicates that the proportion of women suffering from opioid use disorders at the time of delivery more than doubled between 2017 and 2019 (Hirai, Ko, Owens, Stocks, & Patrick, 2021). While the results of research on the effects of perinatal opioid exposure on academic achievement remain inconclusive (Behnke et al., 2013), some studies have reported that children with a history of perinatal exposure to opioids are more likely to have a diagnosis of a learning disorder, such as dyslexia or dyscalculia, and are more likely to require special education services (Levine, Liu & Das, 2008).

A single study of prenatal opioid exposure met inclusion criteria. This study was conducted in the United States and was judged to be of high quality, meeting all but one of the quality indicators. Landi, Avery, Crowley, Wu, and Mayes (2017; n = 110) used EEG to examine the behavioral and neurobiological correlates of language and reading ability between participants who had confirmed prenatal cocaine exposure (PCE) and those who did not (non-drug exposed; NDE). The authors reported that the PCE group showed deficits in reading and language abilities even after controlling for factors such as socioeconomic status and exposure to other substances. Recorded EEG responses indicated atypical event-related potentials in the PCE group related to orthographic-phonological mapping (reduced N1/P2 response) during three reading tasks (word-level rhyme and semantic priming, and sentence-level semantic anomaly), as well as rhyme and semantic processing during language tasks (in the N400 response), suggesting that the impacts of PCE on language and reading skills may persist into adolescence. Notably, this finding is at variance with the conclusions of some behavioral research in the field (e.g. Behnke et al., 2013) while consistent with the conclusions of others (e.g. Bandstra et al., 2001), underscoring the need for additional research in this area.

**S5. Organohalogens**

Organohalogens are a class of organic (carbon-based) chemicals that contain one or more halogen-group (such as chlorine, bromine, or fluorine) atoms. These chemicals are often extremely stable, leading to their use in various industrial applications, as well as their persistence in the environment. Perinatal exposure to organohalogens has been linked to negative impacts on academic achievement. Studies have linked exposure to high levels of organohalogens, such as polychlorinated biphenyls (PCBs) and polybrominated diphenyl ethers (PBDEs), to decrements in cognitive abilities, memory and attention, and academic achievement (Stewart et al., 2008; Zhang et al., 2017), including decrements in reading skills at age eight. These chemicals have also been found to interfere with brain development and disrupt the normal functioning of neurotransmitters, which can in turn affect learning and behavior (Vuong et al., 2020).

A single study of perinatal organohalogen exposure met inclusion criteria and was thus subjected to review. This study was conducted in the United States and was judged to be of high quality, meeting all but one of the quality indicators per research team consensus. Margolis et al. (2020; n = 33) used resting-state fMRI to evaluate the relationship between maternal serum PBDE concentrations and functional connectivity of the reading network in offspring at age five. No association was detected between maternal serum PBDE levels and participant word reading scores; however, higher maternal serum PBDE concentration was associated with reduced global efficiency in the reading network. No relationship was detected between PBDE exposure and the efficiency of the default mode network, providing evidence of the specificity of findings. Moreover, higher efficiency of the reading network was associated with better reading scores in this study as well as others, suggesting that PBDE-related alterations of reading network architecture may underlie downstream effects of prenatal PBDE on reading.

This study’s findings, while modest in scope, are consonant with previous research that has linked PBDE exposure to neurodevelopmental alterations. For example, a large-scale study (n = 210) linked prenatal PBDE exposure to decrements in IQ and reduced performance on several neurodevelopmental measures (Herbstman et al., 2010), while a smaller-scale study (n = 62) detected significant associations between organohalogen exposure and alterations in attention, motor skills, and behavior among school-age children (Roze et al., 2009).

**Table S2**

*Assessment of article quality*

| **First Author, Date** | **Aims described?** | **Hypothesis described?** | **Inclusion criteria described?** | **Consent obtained?** | **Sufficient demographic data reported?** | **SES data reported?** | **Comparability of groups demonstrated?** | **Outcome variables clearly described?** | **Outcomes clearly reported?** | **Data reported consistent with design?** | **Error and/or variability reported?** | **Obtained probability values reported?** |
| --- | --- | --- | --- | --- | --- | --- | --- | --- | --- | --- | --- | --- |
| **Alcohol Studies** | | | | | | | | | | | | |
| Ben-Shachar, 2020 | Yes | Yes | Yes | Yes | Yes | Yes | Yes | Yes | Yes | Yes | Yes | Yes |
| Gautam, 2015 | Yes | Yes | Yes | Yes | No | Yes | Yes | Yes | Yes | Yes | Yes | Yes |
| Glass, 2017 | Yes | Yes | Yes | Yes | Yes | Yes | Yes | Yes | Yes | Yes | Yes | Yes |
| Lebel, 2010 | Yes | Yes | Yes | Yes | No | No | Not Applicable | Yes | Yes | Yes | Yes | Yes |
| Little, 2018 | Yes | Yes | Yes | Yes | Yes | Yes | Not Applicable | Yes | Yes | Yes | Yes | Yes |
| McLachlan, 2020 | Yes | Yes | Yes | Yes | No | Yes | Yes | Yes | Yes | Yes | Yes | Yes |
| Meintjes, 2010 | Yes | Yes | Yes | Yes | Yes | Yes | Yes | Yes | Yes | Yes | Yes | Yes |
| Miles, 2021 | Yes | Yes | Yes | Yes | Yes | Yes | Yes | Yes | Yes | Yes | Yes | Yes |
| Santhanam, 2009 | Yes | Yes | Yes | Yes | No | Yes | No | Yes | Yes | Yes | Yes | Yes |
| Sowell, 2008 | Yes | Yes | Yes | Yes | Yes | No | No | Yes | Yes | Yes | Yes | Yes |
| Treit, 2013 | Yes | Yes | Yes | Yes | Yes | Yes | Yes | Yes | Yes | Yes | Yes | Yes |
| Woods, 2015 | Yes | Yes | Yes | Yes | Yes | Yes | Yes | Yes | Yes | Yes | Yes | Yes |
| Woods, 2018 | Yes | Yes | Yes | Yes | Yes | Yes | Yes | Yes | Yes | Yes | Yes | Yes |
| **Opioid Studies** | | | | | | | | | | | | |
| Landi, 2017 | Yes | Yes | Yes | No | Yes | Yes | Yes | Yes | Yes | Yes | Yes | Yes |
| **Organohalogen Studies** | | | | | | | | | | | | |
| Margolis, 2020 | Yes | Yes | Yes | Yes | No | Yes | Not Applicable | Yes | Yes | Yes | No | Yes |
| **Prematurity Studies** | | | | | | | | | | | | |
| Arhan, 2017 | Yes | Yes | Yes | Yes | No | Yes | Yes | Yes | Yes | Yes | Yes | Yes |
| Allin, 2001 | Yes | Yes | Yes | Yes | No | Yes | Yes | Yes | Yes | Yes | Yes | Yes |
| Anderson, 2017 | Yes | Yes | Yes | Yes | No | Yes | Not Applicable | Yes | Yes | Yes | Yes | Yes |
| Andrews, 2010 | Yes | Yes | No | Yes | No | No | No | Yes | Yes | Yes | Yes | Yes |
| Bäuml, 2017 | Yes | Yes | Yes | Yes | No | Yes | Yes | Yes | Yes | Yes | Yes | Yes |
| Belfort, 2016 | Yes | Yes | Yes | Yes | No | Yes | Not Applicable | Yes | Yes | Yes | Yes | No |
| Bruckert, 2019 | Yes | Yes | No | Yes | No | Yes | Yes | Yes | Yes | Yes | Yes | Yes |
| Cheong, 2013 | Yes | Yes | Yes | Yes | No | Yes | Yes | Yes | Yes | Yes | Yes | Yes |
| Clark, 2017 | Yes | Yes | Yes | Yes | Yes | No | Yes | Yes | Yes | Yes | Yes | Yes |
| Collins, 2019 | Yes | Yes | Yes | Yes | No | Yes | Yes | Yes | Yes | Yes | Yes | Yes |
| Collins, 2021 | Yes | Yes | Yes | Yes | No | Yes | Yes | Yes | Yes | Yes | Yes | Yes |
| Dubner, 2019 | Yes | Yes | Yes | Yes | Yes | Yes | Yes | Yes | Yes | Yes | Yes | Yes |
| Feldman, 2012 | Yes | Yes | Yes | Yes | No | Yes | Yes | Yes | Yes | Yes | Yes | Yes |
| Frye, 2009 | Yes | Yes | Yes | Yes | No | Yes | Yes | Yes | Yes | Yes | Yes | Yes |
| Frye, 2010 | Yes | Yes | Yes | Yes | No | Yes | Yes | Yes | Yes | Yes | Yes | Yes |
| Gozzo, 2009 | Yes | Yes | Yes | Yes | Yes | Yes | Yes | Yes | Yes | Yes | Yes | Yes |
| Isaacs, 2001 | Yes | Yes | Yes | Yes | No | No | Yes | Yes | Yes | Yes | Yes | Yes |
| Kelly, 2016 | Yes | Yes | Yes | Yes | No | No | Yes | Yes | Yes | Yes | Yes | Yes |
| Kesler, 2006 | Yes | Yes | Yes | Yes | No | No | Yes | Yes | Yes | Yes | Yes | Yes |
| Klein, 2014 | Yes | Yes | Yes | Yes | No | No | Not Applicable | Yes | Yes | Yes | Yes | Yes |
| Klein, 2018 | Yes | Yes | Yes | Yes | No | No | Not Applicable | Yes | Yes | Yes | Yes | Yes |
| Ment, 2006 | Yes | Yes | Yes | Yes | Yes | Yes | Yes | Yes | Yes | Yes | Yes | Yes |
| Myers, 2010 | Yes | Yes | Yes | Yes | No | Yes | Yes | Yes | Yes | Yes | Yes | Yes |
| Pavlova, 2009 | Yes | Yes | Yes | Yes | No | No | Yes | Yes | Yes | Yes | Yes | Yes |
| Scott, 2011 | Yes | Yes | Yes | Yes | No | Yes | Yes | Yes | Yes | Yes | Yes | Yes |
| Stewart, 1999 | Yes | Yes | Yes | Yes | No | Yes | Yes | Yes | Yes | Yes | No | Yes |
| Thompson, 2015 | Yes | Yes | Yes | Yes | No | No | No | Yes | Yes | Yes | Yes | Yes |
| Thompson, 2020 | Yes | Yes | Yes | No | No | No | Yes | Yes | Yes | Yes | Yes | Yes |
| Travis, 2015 | Yes | Yes | Yes | Yes | No | Yes | Yes | Yes | Yes | Yes | Yes | Yes |
| Travis, 2016 | Yes | Yes | Yes | Yes | No | Yes | Yes | Yes | Yes | Yes | Yes | Yes |
| Ullman, 2015 | Yes | Yes | Yes | Yes | No | No | Yes | Yes | Yes | Yes | Yes | Yes |
| Van Ettinger-Veenstra, 2017 | Yes | Yes | Yes | Yes | No | No | Yes | Yes | Yes | Yes | Yes | Yes |

**Table S3**

*Summary of studies retained in this systematic review*

| **First Author, Date** | **Summary** |
| --- | --- |
| **Alcohol Studies** |  |
| Ben-Shachar, 2020 | This study investigated the impact of PAE on error detection during arithmetic processing in adolescents. The PAE group demonstrated lower accuracy compared to the typically developing group, with accuracy inversely related to diagnosis severity. EEG results indicated that the TD and heavily exposed nonsyndromal groups showed expected theta-burst activity differentiating correct and incorrect equations, while the FAS/pFAS groups exhibited altered theta reactions to errors, depending on the severity of their diagnosis. These findings suggest that arithmetical error-related theta activity is affected by PAE and may serve as a marker of FASDs. |
| Gautam, 2015 | This study examined white matter and subcortical gray matter volume changes in children with PAE and controls. Children with PAE exhibited smaller volumes across the brain compared to controls. Significant group-score interactions were found in specific brain regions, including temporal and parietal regions for arithmetic scores and frontal and parietal regions for behavioral measures. These findings suggest abnormal brain-behavior relationships in children with PAE and indicate potential opportunities for intervention. |
| Glass, 2017 | This study examined academic achievement in children with heavy PAE and non-exposed controls, revealing deficits across all domains with a relative weakness in math reasoning in the PAE group. Analyses identified several surface area clusters in the brain that were differentially related to math and spelling performance in the PAE group. Specifically, math performance was associated with surface area clusters in the left superior parietal and right lateral/middle occipital regions, while spelling performance was related to clusters in bilateral inferior and medial temporal regions. |
| Lebel, 2010 | This study investigated the relationship between math skills and brain white matter structure in children with FASDs using DTI. Analyses revealed four clusters with significant associations between FA and math scores: two positively correlated clusters in the left parietal region, one positively correlated cluster in the left cerebellum, and one negatively correlated cluster in the bilateral brainstem. Diffusion tractography identified specific white matter tracts passing through these clusters, including the left SLF, left CST and body of the CC, middle cerebellar peduncle, and bilateral projection fibers including the anterior and posterior limbs of the internal capsule. |
| Little, 2018 | This study investigated resting-state functional networks in children and adolescents with FASD compared to healthy controls. Participants with FASD showed significant spatial overlap of positively correlated regions in the six core networks compared to controls, but less overlap for negatively correlated regions. Additionally, lower internetwork connectivity was observed in regions associated with the salience network, frontal-parietal network, and language network in participants with FASD, suggesting deficits in the functional network brain architecture that may be related to cognitive impairment, particularly in mathematics ability and attention. |
| McLachlan, 2020 | This study investigated the impact of SES on brain volumes in 69 individuals with PAE and 70 neurotypical controls. The authors reported significantly smaller brain volumes in all 13 areas measured, and worse cognitive performance across 14 measures in the PAE group, irrespective of SES. While higher SES in the control group correlated with larger hippocampus and amygdala volumes, no such relationship was apparent in the PAE group, suggesting that PAE-related brain injury may be resistant to SES-related remediation. |
| Meintjes, 2010 | This study used fMRI to investigate the neural correlates of number processing in 15 children with FAS or pFAS and 18 matched controls. The control group activated the expected fronto-parietal network during Proximity Judgement and Exact Addition tasks, including the anterior HIPS, left posterior HIPS, left precentral sulcus, and posterior medial frontal cortex. However, the FAS/pFAS group exhibited atypical activation in additional parietal pathways during the Proximity Judgement task and more diffuse activations, including the cerebellar vermis and cortex, during the Exact Addition task, possibly reflecting compensatory activity. |
| Miles, 2021 | This study explored whether smaller IPS volumes contribute to number-processing deficits in 52 children with PAE, aged 9-14. The study reported significantly smaller left medial IPS and left lateral IPS volumes in the FAS/pFAS group compared to controls, even after adjusting for potential confounders and smaller overall brain size. However, while larger bilateral medial IPS volumes correlated with better WISC IQ Arithmetic scores, these regional IPS volume changes did not mediate the effect of PAE on arithmetic performance, indicating that PAE-induced changes in IPS volume do not mediate alcohol-related deficits in arithmetic. |
| Santhanam, 2009 | This study examined the impact of PAE on the neural activation associated with arithmetic processing in young adults. Findings indicated an exposure-dependent response, with dysmorphic PAE individuals demonstrating significantly lower task-related performance and activation in regions known for arithmetic processing, including left superior and right inferior parietal regions and medial frontal gyrus. The authors conclude that physical dysmorphia might serve as an indicator functional damage to regions associated with arithmetic calculation. |
| Sowell, 2008 | This study reported lower white matter integrity in the brains of people with fetal alcohol spectrum disorders FASDs, particularly in the right lateral temporal lobe and both sides of the CC’s splenium. These abnormalities, indicated by lower FA, suggest disorganized fiber tracts or less myelin due to prenatal alcohol exposure. In the FASD group, poorer visuomotor integration performance significantly correlated with these FA abnormalities in the splenium. |
| Treit, 2013 | This study investigated brain development trajectories in children with FASD and typically-developing controls using longitudinal DTI and T1-weighted volumetric MRI. The FASD group demonstrated reduced total brain, white, cortical gray, and deep gray matter volumes as well as altered developmental progression in the SLF and superior and inferior fronto-occipital fasciculus, marked by a larger reduction in MD, which correlated with improvements in language scores. |
| Woods, 2015 | This study investigated the effects of PAE on functional activity in parietal regions during number processing tasks in 49 children. Despite similar behavioral performance, children with greater PAE demonstrated less activation in the anterior section of the right HIPS (a region known for quantity representation and manipulation) during simple addition and magnitude comparison tasks. Children with FAS/pFAS showed possibly compensatory increased activation of the AG during magnitude comparison tasks, suggesting alternative neural strategies for number processing. |
| Woods, 2018 | This study investigated the impact of PAE on the neural correlates of non-symbolic number comparison and numerical distance effect in children. Despite no group differences in performance, control children activated the right PSPL and the right HIPS more than exposed children during the non-symbolic number comparison task. More heavily exposed children recruited the left AG more with increasing task difficulty, possibly compensating for impairments in PSPL and IPS function. |
| **Opioid Studies** |  |
| Landi, 2017 | This study compared older adolescents with prenatal cocaine exposure (PCE) to nondrug-exposed (NDE) adolescents on cognitive and linguistic assessments, revealing deficits in reading and language abilities even after controlling for socioeconomic status and exposure to other substances. ERP data further demonstrated atypical orthography to phonology mapping (reduced N1/P2 response) and atypical rhyme and semantic processing (N400 response) in the PCE group, indicating continued impact on language and reading skills into late teenage years. |
| **Organohalogen Studies** |  |
| Margolis, 2020 | This study examined the associations between prenatal PBDE exposure and functional connectivity of a reading-related network (RN) in 5-year-old children using resting-state fMRI. Maternal serum PBDE concentrations were measured during gestation, and higher PBDE levels were associated with reduced efficiency and integration of the RN, but not with the default mode network, suggesting specificity of the findings. The altered RN efficiency may underlie associations between PBDE exposure and reading problems observed in older children. |
| **Prematurity Studies** |  |
| Arhan, 2017 | This study examined brain volumes and cognitive outcomes in 22 low-risk preterm children compared to matched term controls. Volumetric MRI analyses at age nine revealed significant reductions in cerebellum, hippocampus, and corpus callosum regions among preterm children. Likewise, cognitive tests indicated significantly lower performance in the preterm group. Notably, a significant association was found between reduced cerebellar volumes and lower attention and executive function scores, and a significant association was found between CC measures and Arithmetic performance. |
| Allin, 2001 | This study used volumetric analysis of brain MRI scans to examine potential cerebellum abnormalities in adolescents born VPT (<33 weeks' gestation). Results indicate these individuals had significantly reduced cerebellar volume compared to term-born controls. This reduced cerebellar volume correlated significantly with various cognitive test scores, including the Wechsler Intelligence Scale for Children-Revised, the Kaufman Assessment Battery for Children, and the Schonnel reading age, suggesting cerebellar abnormalities may contribute to the cognitive deficits found in very pre-term born individuals. |
| Anderson, 2017 | This study assessed the connection between newborn MRI brain abnormalities and neurodevelopmental impairment at age 7 in VPT children. The authors reported that higher global brain, cerebral white matter, and deep gray matter abnormality scores corresponded to poorer outcomes in IQ, spelling, math computation, and motor function. Moreover, higher cerebellum abnormality scores were linked to poorer IQ, math computation, and motor outcomes, suggesting that brain MRI at term equivalent can predict achievement outcomes independent of clinical and social factors. |
| Andrews, 2010 | This study used dMRI to examine the relationship between white matter integrity and reading ability in a cohort of 28 children, including 19 preterm children. The authors reported that preterm children had lower reading scores across all tested subtests and significant correlations existed between birthweight and FA in the whole CC, and between reading skill and FA in the genu and body of the CC. These results suggest that perinatal white-matter injury of the central CC may affect long-term reading performance. |
| Bäuml, 2017 | This study investigated the relationship between childhood mathematic abilities and long-term neurocognitive outcomes in PT individuals compared to term-born individuals. The researchers reported that childhood mathematic abilities were more strongly associated with adult IQ in PT individuals, even when controlling for IQ at age eight. Additionally, the association between childhood mathematic abilities and adult fronto-parietal intrinsic functional connectivity was altered in preterm-born individuals, suggesting distinct functional roles of these intrinsic networks in relation to mathematic abilities for preterm individuals. |
| Belfort, 2016 | This study investigated the impact of breast milk intake in the first 28 days of life on neurological outcomes in VPT infants. The researchers studied 180 infants born at <30 weeks' gestation or <1250 grams birth weight and measured the number of days they received >50% of enteral intake as breast milk. The results indicated that a greater number of days with >50% breast milk intake was associated with larger deep nuclear gray matter volume at term equivalent age and improved performance on IQ, mathematics, working memory, and motor function tests at 7 year in VPT infants. However, no differences in regional brain volumes at 7 years were observed in relation to breast milk intake. |
| Bruckert, 2019 | This study investigated the association between white matter properties at age 6 years and reading outcome at age 8 years in children born PT compared to children born FT). dMRI and standardized measures of non-verbal IQ, language, and phonological awareness were obtained at age 6 for both PT (n=34) and FT children (n=37). The analysis focused on several white matter pathways, including the left Arc, bilateral SLF, and left ICP, which were previously shown to predict reading outcome in FT children. The results indicated that the prediction of reading outcome based on mean tract-FA at age 6 years was moderated by birth group status, with microstructural properties of these cerebral and cerebellar pathways predicting later reading outcome in FT but not in PT children. |
| Cheong, 2013 | This study compared brain volumes of multiple tissues and structures between extremely preterm (EP) and term-born control adolescents. EP adolescents demonstrated smaller brain volumes, particularly in the thalamus and hippocampus, and had lower IQ and poorer educational skills compared to controls. Total brain tissue volume explained 20-40% of the cognitive and educational outcome differences between EP and controls, indicating that smaller brain size is a significant contributor to the underperformance of EP adolescents. |
| Clark, 2017 | This study investigated the neural correlates of mathematics learning disabilities in individuals born PT (< 37 weeks GA). Both PT and FT groups showed activation in superior and inferior frontal and parietal regions, associated with numeric processing, during a magnitude comparison task. However, the PT group exhibited increased signal change in right inferior frontal and parietal regions during non-symbolic magnitude comparison, which was associated with poorer performance on a calculation task. These findings suggest that healthy, high-functioning adults born PT may rely more on fronto-parietal networks when processing non-symbolic magnitudes. |
| Collins, 2019 | This study examined 114 individuals born VPT (< 32 weeks' gestational age) and 36 individuals born FT at 13 years old to explore the associations between mathematics and reading performance with white matter microstructure. Using DTI and advanced diffusion modelling techniques, the researchers reported that both mathematics and reading performance were linked to white matter microstructure in specific brain regions in VPT and FT children. Furthermore, the association of mathematics and reading performance with white matter microstructure in VPT children varied based on the presence of impairment. |
| Collins, 2021 | This study examined the associations between white matter maturation and math computation ability in children born VPT (<32 weeks' gestation) and FT peers. They reported that higher white matter fiber density, fiber-bundle cross-section, and combined fiber density and cross-section in specific white matter tracts were associated with better math computation ability at 7 and 13 years in both VPT and FT children. Additionally, accelerated maturation of the posterior body of the CC was linked to greater math computation development over time. These findings indicate that white matter maturation is a significant factor in math computation ability during late childhood, regardless of birth group. |
| Dubner, 2019 | This study compared PT children with and without neonatal inflammatory conditions (PT+ and PT-) to FT children regarding white matter microstructure and cognitive outcomes at 6 years old. PT+ children demonstrated lower FA and higher MD in multiple segments of the CC compared to PT- and FT groups. Additionally, executive function scores were less favorable in PT+ children, and occipital FA was significantly correlated with IQ, reading, and executive function measures across the entire sample, while anterior frontal and superior parietal FA were significantly correlated with executive function. These findings suggest that neonatal inflammation is a factor contributing to variations in long-term neurobiological and neuropsychological outcomes in PT children. |
| Feldman, 2012 | This study examined the association between white matter properties and language and reading skills in PT and FT children. DTI was used to analyze FA in both groups. In the PT group, several regions of the white matter skeleton were significantly associated with verbal IQ, linguistic processing speed, syntactic comprehension, and decoding. The findings suggest that higher performance in language and reading skills in PT children is linked to higher FA of a bilateral and distributed white matter network, whereas no such associations were found in the full-term group. |
| Frye, 2009 | This study used MEG to investigate cortical reorganization related to prematurity in adolescents. Participants were selected based on gestational age, birth weight, neonatal complications, and reading ability. Adolescents born at high-risk demonstrated a greater NOD in the left prefrontal area compared to those born at low-risk and term during both real-word and non-word rhyme tasks, indicating increased prefrontal cortical activation. These findings suggest a reorganization of the prefrontal cortex in adolescents born PT. |
| Frye, 2010 | This study explored the relationship between cognitive abilities/achievement and white-matter structure in adolescents born PT without obvious brain injury. The researchers analyzed FA, RD, and volume of three major white-matter fasciculi in thirty-two adolescents. They found that left-hemisphere SLF FA and RD were linked to reading-related skills, while right-hemisphere SLF FA was related to attention skills. Additionally, SLF volume decreased as cognitive skills declined for PT adolescents, suggesting the presence of cryptic white-matter injury, possibly related to oligodendrocyte or axonal loss, despite normal clinical neuroimaging. |
| Gozzo, 2009 | This study investigated cerebral connectivity differences during an auditory language task between preterm PT and FT school-age children using fMRI. PT children demonstrated significantly stronger neural circuits between Wernicke's area and the right inferior frontal gyrus (Broca's area homologue), as well as both left and right supramarginal gyri (components of the inferior parietal lobules) compared to term controls, indicating a delay in maturation of neural networks or the engagement of alternate circuits for language processing in PT subjects. |
| Isaacs, 2001 | This study investigated the neural basis of learning difficulties in VPT adolescents born at 30 weeks’ gestation or less, focusing on arithmetic calculation deficits. Using voxel-based morphometry, the authors reported that neurologically normal VPT children without deficits in calculation ability showed greater grey matter in the left parietal lobe compared to those with calculation deficits. This finding provide evidence of a structural neural correlate for calculation ability in neurologically normal VPT individuals. |
| Kelly, 2016 | This study explored the impact of VPT birth (<32 weeks' gestation) on brain white matter structure and its correlation with perinatal factors and neurodevelopmental outcomes. The researchers used NODDI and FA to compare white matter characteristics between 145 VPT and 33 control children aged 7 years. Results showed that VPT children had lower FA and higher axon dispersion within several major white matter tracts compared to full-term children. Moreover, changes in FA, axon dispersion, and axon density were associated with poorer neurodevelopmental outcomes in VPT children. |
| Kesler, 2006 | This study examined cortical gyrification in PT and FT control children at 8 years of age and its association with language ability. PT children demonstrated increased gyrification in bilateral temporal lobes compared to term controls. The left temporal gyrification index showed negative correlations with left temporal lobe gray matter volume and reading recognition scores in the preterm group, indicating differential vulnerability of cortical development in the temporal lobe due to preterm birth. |
| Klein, 2014 | This study investigated neural correlates of intentional and automatic number processing in 6- and 7-year-old children born PT. Behavioral findings demonstrated significant numerical distance and size congruity effects. Imaging results demonstrated overlapping fronto-parietal activation for intentional and automatic number processing, a shift of activation from frontal to parietal regions based on gestational age and birth weight, and task-specific association between math proficiency and fMRI signal in distinct parietal lobe regions, suggesting commonalities and specificities in intentional and automatic number processing. |
| Klein, 2018 | This study investigated the neural correlates of number processing in 6- and 7-year-old children born PT and found that gestational age predicted the frontal-to-parietal shift of activation observed during intentional number magnitude processing. The numerical distance effect elicited the typical frontoparietal activation pattern, while the size congruity effect was associated with brain areas related to cognitive control. These findings suggest that human numerical development may start before birth, and prematurity could hinder neural facilitation of the brain circuitry involved in numerical cognition. |
| Ment, 2006 | This study used fMRI to examine the effects of neonatal indomethacin treatment on brain activation during a language task. Significant differences in cognitive and achievement test scores were detected between the PT and FT children. The study reported a significant treatment-by-gender effect during phonological processing in three brain regions: the left inferior parietal lobule, the left inferior frontal gyrus (Broca's area), and the right dorsolateral prefrontal cortex, suggesting that indomethacin administration influences neural development differently in male and female preterm infants. |
| Myers, 2010 | This study investigated language deficits in 31 PT adolescents compared to 36 FT controls using an fMRI passive language task and various neurodevelopmental assessments. PT participants scored significantly lower on all components WISC-III, but there was no significant difference in PPPVT-R scores between the groups. The study reported increased functional connectivity between Wernicke's area and the right supramarginal gyrus in PT participants, suggesting a reliance on an alternate language pathway in PT adolescents. |
| Pavlova, 2009 | This study investigated the relationship between calculation abilities and the extent and topography of periventricular lesions in adolescents born prematurely. Findings suggested that adolescents with periventricular leukomalacia (PVL) demonstrated lower performance on mental calculation tasks than both preterm and term-born peers without PVL. However, calculation abilities did not correspond with the volumetric extent or topography of lesions in either brain hemisphere. |
| Scott, 2011 | This study investigated the association between brain structure and language and executive functioning in VPT adolescents compared to FT controls. The authors reported differential associations between spelling scores and grey matter volume in the frontal regions in the VPT and control groups, and among males and females, suggesting that VPT birth might alter the typical structure-function relationship concerning spelling abilities. |
| Stewart, 1999 | This study examined the long-term neurocognitive and behavioral impact of VPT birth (<33 weeks) on adolescents, and the associated changes in brain structure. The authors reported that more than half of the PT individuals had abnormal MRI brain scans in adolescence, with common abnormalities in the ventricles, CC, and white matter, which were linked to reading, adjustment, and neurological impairments. |
| Thompson, 2015 | This study investigated the size, microstructure, and development of the CC in VPT and FT children from infancy to age 7. The authors reported that VPT children presented smaller posterior CC regions, higher diffusivity, and lower FA at age 7 compared to FT peers, and they experienced faster reduction in diffusivity over time. The findings also indicated that CC microstructural abnormalities and slower CC development in VPT children correlated with motor dysfunction, poorer math skills, and poorer visual perception. |
| Thompson, 2020 | This study investigated the relationship between VPT birth (<32 weeks' gestation) and corticostriatal and thalamocortical tract connectivity in children at age 7. Very preterm children exhibited reduced connectivity in tracts linking the caudate to right motor areas and the thalamus with left motor areas compared to term-born controls. Lower connectivity in these tracts correlated with poorer motor functioning and, in controls, lower reading performance, suggesting that these tracts' vulnerabilities contribute to the motor impairments observed in very preterm children. |
| Travis, 2015 | This study investigated the role of cerebellar white matter pathways in major reading component skills among FT and PT children and adolescents. Analyses indicated that FA of the cerebellar peduncles correlated significantly with decoding and reading comprehension measures, with negative correlations in the superior and inferior cerebellar peduncles and a positive correlation in the middle cerebellar peduncle. Distinct reading sub-skills also correlated with FA in different cerebellar peduncles, suggesting the first associations between cerebellar peduncles' microstructure and reading component skills. |
| Travis, 2016 | This study explored the link between white matter diffusion properties and reading skills in children and adolescents, both PT and FT. The authors reported that FA  in the anterior SLF, Arc, CST), and uncinate fasciculus correlated with single word reading and comprehension in both groups. While this correlation was negative in FT individuals, PT individuals showed a positive correlation, suggesting divergent neurobiological mechanisms between these populations. |
| Ullman, 2015 | This study used neonatal s/dMRI measures to examine the neurostructural and white matter correlates of math skills in preterm children at 5 and 7 years of age. Results showed localized regions around the insula and putamen in the neonatal Jacobian map that positively correlated with early math skills in preterm children at both ages. Additionally, neonatal FA was positively associated with working memory and early math skills at five years, highlighting potential brain markers for early identification of preterm children at risk for cognitive and academic impairment. |
| Van Ettinger-Veenstra, 2017 | This study investigated neural activation related to components of reading comprehension in young very low birth weight (VLBW) adolescents compared to normal birth weight peers using fMRI. The VLBW group demonstrated increased phonological activation in left inferior frontal gyrus, decreased orthographic activation in right supramarginal gyrus, and decreased semantic activation in left inferior frontal gyrus. These findings suggest that VLBW adolescents may employ compensatory mechanisms by recruiting additional brain regions. |

**References [Studies included in review bolded.]**

Bandstra, E. S., Morrow, C. E., Anthony, J. C., Accornero, V. H., & Fried, P. A. (2001). Longitudinal investigation of task persistence and sustained attention in children with prenatal cocaine exposure. *Neurotoxicology and Teratology*, *23*(6), 545–559. <https://doi.org/10.1016/s0892-0362(01)00181-7>

Behnke, M., Smith, V. C., Committee on Substance Abuse, & Committee on Fetus and Newborn (2013). Prenatal substance abuse: short- and long-term effects on the exposed fetus. *Pediatrics*, *131*(3), e1009–e1024. <https://doi.org/10.1542/peds.2012-3931>

Button, K. S., Ioannidis, J. P., Mokrysz, C., Nosek, B. A., Flint, J., Robinson, E. S., & Munafò, M. R. (2013). Power failure: why small sample size undermines the reliability of neuroscience. *Nature Reviews. Neuroscience*, *14*(5), 365–376. <https://doi.org/10.1038/nrn3475CDC>

Cremers, H. R., Wager, T. D., & Yarkoni, T. (2017). The relation between statistical power and inference in fMRI. *PloS one*, *12*(11), e0184923. <https://doi.org/10.1371/journal.pone.0184923>

Durnez, J., Degryse, J., Moerkerke, B., Seurinck, R., Sochat, V., Poldrack, R. A., & Nichols, T. E. (2016). Power and sample size calculations for fMRI studies based on the prevalence of active peaks. *BioRxiv*, 049429. <https://doi.org/10.1101/049429>

Herbstman, J. B., Sjödin, A., Kurzon, M., Lederman, S. A., Jones, R. S., Rauh, V., Needham, L. L., Tang, D., Niedzwiecki, M., Wang, R. Y., & Perera, F. (2010). Prenatal exposure to PBDEs and neurodevelopment. *Environmental Health Perspectives*, *118*(5), 712–719. <https://doi.org/10.1289/ehp.0901340>

Hirai, A. H., Ko, J. Y., Owens, P. L., Stocks, C., & Patrick, S. W. (2021). Neonatal abstinence syndrome and maternal opioid-related diagnoses in the US, 2010-2017. *JAMA, 325*(2), 146. <https://doi.org/10.1001/jama.2020.24991>

**Landi, N., Avery, T., Crowley, M. J., Wu, J., & Mayes, L. (2017). Prenatal Cocaine Exposure Impacts Language and Reading Into Late Adolescence: Behavioral and ERP Evidence. *Developmental Neuropsychology*, *42*(6), 369-386.** [**https://doi.org/10.1080/87565641.2017.1362698**](https://doi.org/10.1080/87565641.2017.1362698)

Levine, T. P., Liu, J., Das, A., Lester, B., Lagasse, L., Shankaran, S., Bada, H. S., Bauer, C. R., & Higgins, R. (2008). Effects of prenatal cocaine exposure on special education in school-aged children. P*ediatrics*, 122(1), e83–e91. <https://doi.org/10.1542/peds.2007-2826>

Lindquist, M. A., & Mejia, A. (2015). Zen and the art of multiple comparisons. *Psychosomatic Medicine*, *77*(2), 114–125. <https://doi.org/10.1097/PSY.0000000000000148>

**Margolis, A. E., Banker, S., Pagliaccio, D., De Water, E., Curtin, P., Bonilla, A., Herbstman, J. B., Whyatt, R., Bansal, R., Sjödin, A., Milham, M. P., Peterson, B. S., Factor-Litvak, P., & Horton, M. K. (2020). Functional connectivity of the reading network is associated with prenatal polybrominated diphenyl ether concentrations in a community sample of 5 year-old children: A preliminary study. *Environment International*, *134*, 105212.** [**https://doi.org/10.1016/j.envint.2019.105212**](https://doi.org/10.1016/j.envint.2019.105212)

Mumford, J. A. (2012). A power calculation guide for fMRI studies. *Social cognitive and affective neuroscience*, *7*(6), 738-742.

<https://doi.org/10.1093/scan/nss059>

Roiser, J. P., Linden, D. E., Gorno-Tempinin, M. L., Moran, R. J., Dickerson, B. C., & Grafton, S. T. (2016). Minimum statistical standards for submissions to Neuroimage: Clinical. *NeuroImage. Clinical*, *12*, 1045–1047. https://doi.org/10.1016/j.nicl.2016.08.002

Roze, E., Meijer, L., Bakker, A., Van Braeckel, K. N., Sauer, P. J., & Bos, A. F. (2009). Prenatal exposure to organohalogens, including brominated flame retardants, influences motor, cognitive, and behavioral performance at school age. *Environmental health perspectives*, *117*(12), 1953–1958. https://doi.org/10.1289/ehp.0901015

Stewart, P. W., Lonky, E., Reihman, J., Pagano, J., Gump, B. B., & Darvill, T. (2008). The relationship between prenatal PCB exposure and intelligence (IQ) in 9-year-old children. *Environmental Health Perspectives*, *116*(10), 1416-1422.

Turner, B.O., Paul, E.J., Miller, M.B. *et al.* Small sample sizes reduce the replicability of task-based fMRI studies. *Commun Biol* **1**, 62 (2018). https://doi.org/10.1038/s42003-018-0073-z

United States Centers for Disease Control and Prevention. (2016, January 1). [CDC press release on alcohol consumption during pregnancy]. CDC. <https://www.cdc.gov/media/releases/2015/p0924-pregnant-alcohol.html>

Vuong, A. M., Yolton, K., Cecil, K. M., Braun, J. M., Lanphear, B. P., & Chen, A. (2020). Flame retardants and neurodevelopment: An updated review of epidemiological literature. *Current Epidemiology Reports*, *7*, 220-236.

Zhang, H., Yolton, K., Webster, G. M., Sjödin, A., Calafat, A. M., Dietrich, K. N., Xu, Y., Xie, C., Braun, J. M., Lanphear, B. P., & Chen, A. (2017). Prenatal PBDE and PCB Exposures and Reading, Cognition, and Externalizing Behavior in Children. *Environmental health perspectives*, *125*(4), 746–752. https://doi.org/10.1289/EHP478
